# Synthetic Strategies and Computational Inhibition Activity Study for Triazinyl-Substituted Benzenesulfonamide Conjugates with Polar and Hydrophobic Amino Acids as Inhibitors of Carbonic Anhydrases

**DOI:** 10.3390/ijms21103661

**Published:** 2020-05-22

**Authors:** Mária Bodnár Mikulová, Dáša Kružlicová, Daniel Pecher, Claudiu T. Supuran, Peter Mikuš

**Affiliations:** 1 Department of Pharmaceutical Analysis and Nuclear Pharmacy, Faculty of Pharmacy, Comenius University in Bratislava, Odbojárov 10, SK-832 32 Bratislava, Slovakia; mikulova43@uniba.sk (M.B.M.); kruzlicova@fpharm.uniba.sk (D.K.); pecher1@uniba.sk (D.P.); 2 Toxicological and Antidoping Center, Faculty of Pharmacy, Comenius University in Bratislava, Odbojárov 10, SK-832 32 Bratislava, Slovakia; 3 Neurofarba Department, Section of Pharmaceutical and Nutriceutical Sciences, University of Florence, 50139 Florence, Italy; claudiu.supuran@unifi.it

**Keywords:** triazine, benzenesulfonamide, human carbonic anhydrase, hypoxia, amino acids, inhibition

## Abstract

Various sulfonamide derivatives are intensively studied as anticancer agents owing to their inhibitory activity against human tumor-associated carbonic anhydrase isoforms. In this work, different synthetic procedures for the series of 1,3,5-triazinyl-aminobenzenesulfonamide conjugates with amino acids, possessing polar uncharged, negatively charged, and hydrophobic side chain, were studied and optimized with respect to the yield/purity of the synthesis/product as well as the time of synthetic reaction. These procedures were compared to each other via characteristic HPLC-ESI-DAD/QTOF/MS analytical product profiles, and their benefits as well as limitations were discussed. For new sulfonamide derivatives, incorporating s-triazine with a symmetric pair of polar and some less-polar proteinogenic amino acids, inhibition constants (K_I_s) against four human carboanhydrases (hCAs), namely cytosolic hCA I, II, transmembrane hCA IV, and the tumor-associated, membrane-bound hCA IX isoforms, were computationally predicted applying various methods of the advanced statistical analysis. Quantitative structure-activity relationship (QSAR) analysis indicated an impressive K_I_ ratio (hCA II/hCA IX) 139.1 and hCA IX inhibition constant very similar to acetazolamide (K_I_ = 29.6 nM) for the sulfonamide derivative disubstituted with Gln. The derivatives disubstituted with Ser, Thr, and Ala showed even lower K_I_s (8.7, 13.1, and 8.4 nM, respectively).

## 1. Introduction

Human carbonic anhydrase IX (hCA IX) is a zinc metalloenzyme with extracellular active site catalyzing reversible hydration of carbon dioxide to regulate the acid-base balance. hCA IX differs from other hCA isozymes in several properties. The main structural difference is a presence of the N-terminal proteoglycan-like (PG) domain in the extracellular protein part [1,2]. The PG domain plays a substantial role in adhesion, proliferation, and cell invasion under hypoxia and acidosis microenvironment conditions often contained in solid tumors [3,4]. Hypoxic tumor microenvironment is upregulated by hypoxia-inducible factor 1 (HIF) that activates a gene expression of mediators in various enzymatic pathways and angiogenesis such as glucose transporters, vascular endothelial growth factor as well as hCA IX [5,6]. Many studies and data have established and demonstrated the hCA IX overexpression in various human hypoxic tumor types, such as ductal carcinoma in situ [7]; head and neck squamous cell [8], non-small cell lung [9], ovarian [10], and bladder [11] cancers; cervical [12] and nasopharyngeal [13] carcinomas, brain tumors [14], liver metastases in colorectal cancer [15], etc. Moreover, the hCA IX expression in tumors may occur as a consequence of inactivating process of pVHL (von Hippel-Lindau) tumor suppressor mutation [3], what is represented primarily by the clear renal cell carcinoma [16,17]. On the contrary, hCA IX is only rarely expressed in normal tissues (such as the gastric mucosa, pancreas, and intestine) [18], therefore it can be considered to be a highly selective tumor marker.

Due to the close relation of hCA IX to a wide scale of the oncological diseases, a suppression or inhibition of hCA IX catalytic activity or aiming its PG domain has become a broadly studied, significant, and valuable target in tumor therapy and molecular imaging. Many compounds, namely sulfonamides, sulfamates, coumarins, carbamates, etc., have been demonstrated as highly potent hCA inhibitors with an enhanced selectivity towards the tumor-associated hCA IX isoform, as reviewed in excellent works of Supuran [6,19] or Pastorek and Pastorekova [3,20], and presented also in other recent research papers [21,22,23,24,25,26,27,28,29,30].

However, the great effort is still made in an additional enhancement of the selectivity and potency of hCA inhibitors (hCAi) with antitumor effects. Among them, a large number of small sulfonamide-type molecules have been synthesized and determined as potential hCA XI and hCA XII inhibitors, for example orthanilamide, homosulfonilamide, 4-carboxy-benzenesulfonamide, 1-naphthalenesulfonamide, 1,3-benzenedisulfonamide derivatives, 1,3,4-thiadizole-2-sulfonamide [31], sulfonamides incorporating 1,2,4-triazine substituted with methylamine, water, aliphatic alcohols [32], hydrazine moieties [33], and fluorescent sulfonamides [34]. These structures provided a proper basis for further hCAi designing. Supuran et al. comprehensively studied 1,3,5-triazinyl-substituted benzenesulfonamides incorporating amino acyl/hydroxyalkyl-amino moieties as potential inhibitors of transmembrane isoforms hCA IX, XII, and XIV [35,36]. This series employed a nucleophilic substitution of the chlorine atoms on 1,3,5-triazine in a DIPEA/DMF environment with various nucleophiles (including amino acids Gly, Ala, β-Ala, and Ser) to provide a majority of monosubstituted derivatives [35]. In another paper, the same group substituted one fluorine in 1,3,5-triazinyl-benzenesulfonamide precursor with amines, amino alcohols, amino acids, or amino acid esters in DIPEA/DMF environment. The resulting inhibition constants (K_I_s) related to hCA IX ranged in the interval of 21.8–22.3 nM and for the bulkier substituents in 153–228 nM [37]. Next year the same group implemented 4-amino benzene sulfonamide, ammonia, anilines, morpholine, tert-butylamine, N-methyl-2-amino ethanol, and 1,3-diaminopropane nucleophiles into the 1,3,5-triazinyl-benzenesulfonamide precursor. K_I_s (hCA IX) of the derivatives were in the range of 2.4–34.1 nM [38]. Lolak et al. [39] reported novel ureido benzenesulfonamides incorporating 1,3,5-triazine moieties substituted with aromatic amines on one side and with dimethylamine, morpholine or piperidine on the other side. K_I_s (hCA IX) of the derivatives were in the range of 11.8–184.8 nM. Havránková et al. [40] employed a DMF/K_2_CO_3_ environment for the preparation of a series of substituted s-triazines containing sulfanilamide, homosulfanilamide, or 4-aminoethyl-benzenesulfonamide, and monosubstituted, in the second step, with piperazine as well as aminoalcohol motifs. K_I_s (hCA IX) of the derivatives ranging in the interval of 0.4–307.7 nM. Subsequently, the authors improved this synthetic procedure via Ullmann Cu(I)-catalysis [41]. Mikuš et al. are focused on a systematic study of 1,3,5-triazinyl-substituted benzenesulfonamides incorporating proteinogenic as well as non-proteinogenic (other naturally occurring, synthetic) amino acids in various configurations (mono as well as disubstitution on triazine, pair of equivalent or different substituents). Firstly, they optimized synthetic conditions for triazinyl-benzenesulfonamide conjugates disubstituted with amino acids possessing hydrophobic side chain [42]. The authors demonstrated an improvement of the synthetic procedure for such group of the derivatives when replacing an organic (DIPEA/DMF) environment with a water-based one (sodium carbonate). In this way, several times higher product yields were easily obtained. In their subsequent work, they successfully utilized the same synthetic strategy for the preparation of a new series of triazinyl-substituted aminoalkylbenzenesulfonamides incorporating a symmetric pair of amino acids possessing hydrophobic or negatively charged side chain (β-Ala, Val, Leu, Ile, Met, Phe, Glu, Asp, plus Gly, and Pro) [43]. K_I_s (hCA IX) of the derivatives with non-polar (plus Gly and Pro) and acidic amino acids were in the range of 8.4–2592 nM and 25.8–2364 nM, respectively.

An aim of the present work, considering the promising inhibitory activities and selectivities among the above mentioned amino acid-derivatives against hCA IX, was to prepare 1,3,5-triazinyl-substituted benzenesulfonamides incorporating a symmetric pair of the proteinogenic amino acids that have not been studied in this context so far (i.e., the whole group of polar neutral amino acids and remaining hydrophobic ones). Various water and organic based synthetic procedures were tested and compared for a maximum yield and purity of the products, and optimum synthetic strategies for the groups of derivatives differing in polarity were proposed. New sulfonamide derivatives, prepared by optimized synthetic procedures, were evaluated by QSAR analysis to estimate their inhibition constants (K_I_s) against to four hCA isoforms (hCA I, II, IV, and hCA IX) and inhibition selectivity expressed as K_I_ ratio of the cytosolic hCA II and tumor-associated, membrane-bound hCA IX isoforms (hCA II/hCA IX).

## 2. Results and Discussion

### 2.1. Chemistry

Three amino acids possessing a hydrophobic side chain (Ala, Tyr, Trp) and four amino acids with a polar uncharged chain (Gln, Asn, Ser, Thr) were included into this work to prepare new 1,3,5-triazinyl-substituted benzenesulfonamide derivatives as potential hCA inhibitors. An implementation of polar amino acids, however, can generate new/special demands on the synthetic conditions in comparison with those employed for non-polar amino acids in our previous work [43]. Therefore, a comprehensive study of various synthetic strategies for the derivatives differing in the polarity and charge of the implemented amino acid was another substantial aim of this work. To have more complex data-matrix for such synthetic study, besides the derivatives with new amino acids, some previously published derivatives with acidic (Asp, Glu) and hydrophobic (Phe) amino acids were included too. In this case, also an impact of charge on the synthesis (e.g., via comparing Gln vs. Glu or Asn vs. Asp) or a homology group (Phe, Tyr, Trp) can be investigated in detail. The products of different synthetic procedures were characterized via their HPLC-ESI-DAD/QTOF/MS analytical products profiles, indicating an optimum synthetic strategy for given derivatives/groups of derivatives. The properly purified products were finally characterized by IR, NMR, and MS spectra.

**Figure 1:**
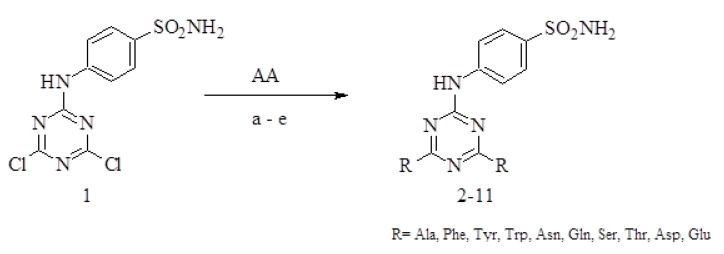
Synthesis of triazinyl-substituted benzenesulfonamide derivatives (**2–11**) using various reaction conditions: (**a**) Na_2_CO_3_ in water, 100 °C; (**b**) NaHCO_3_ in water, 100 °C; (**c**) 0.2% triethylamine (TEA) in water or DMF, reflux; (**d**) THF with Na_2_CO_3_, 66 °C; (**e**) DMF with KF or Na_2_CO_3_ as catalysts, reflux. AA = amino acid.

A series of 1,3,5-triazinyl-substituted benzenesulfonamide derivatives containing a symmetric pair of the amino acids was prepared by the nucleophilic substitution of the dichloro-substituted triazinyl-benzenesulfonamide **1** with three or four equivalents of amino acid (AA) using various synthetic procedures (Figure 1). Different reaction conditions were tested to obtain high purity and yield of desired products (relevant data for crude solid products, demonstrating usefulness of these investigated synthetic strategies, are summarized in Table 1; spectral information for chromatographically purified solid products are in the Sections S1–S10 of the Supplementary Material and corresponding numeric data in the Section 3.2.2).

*Sodium carbonate water-based synthetic procedure.* The sodium carbonate water-based conditions were favorable for a majority of hydrophobic as well as two acidic derivatives [43], but they have not been applied for any polar neutral derivatives so far. The application of Na_2_CO_3_/water environment provided very satisfactory results for the crude products with hydrophobic AA (Ala **2**, Phe **3**, and Trp **5**), expressed via high yields and purities ranging in the intervals of 94–99% and 92.55–96.81%, respectively (see Table 1a). No impurities represented by the mono- and dihydroxy-substituted triazinyl derivatives were formed in the products **2**, **3**, **5** that was confirmed by the HPLC-DAD-MS and NMR analysis. Relatively good yields (73–93%) and purities (87.09–94.42%), according to HPLC-DAD and NMR, were achieved also for the products with polar neutral AA Ser **8**, Thr **9** (see Table 1b) and acidic AA Asp **10**, Glu **11** (see Table 1c). In these polar products, however, 1.50–5.37% of hydroxy-AA-substitued derivatives were detected. Moreover, the HPLC-DAD-MS analysis showed an unknown impurity (in amount of 3–4%), with the *m/z* value of ~438.1 and the proposed summary formula C_15_H_15_N_7_S_2_O_5_, in a majority of the products. The sodium carbonate water-based conditions were less suitable (yields and purities ranged in the intervals of 78–97% and 21.58–49.66%, respectively) only for the products with Tyr **4**, Asn **6**, and Gln **7**. In case of Tyr **4** it can be due to tyrosine sensitivity to oxidation.

*Sodium bicarbonate water-based synthetic procedure*. Sodium bicarbonate as a weaker base was employed instead of sodium carbonate to obtain higher yields and purities of desired products. Indeed, an apparent improvement in the product purity and, hence, final yield was achieved for the conjugates containing polar AA with neutral side chains (Asn **6**, Gln **7**, Ser **8**). An additional great advantage of the bicarbonate synthetic procedure over the carbonate one is a significant shortening of the time of synthesis (more than in half). From the practical point of view, both the bicarbonate as well as carbonate water media are equally suitable for the conjugates with Thr **9**, Phe **3**, and Trp **5**. The synthesis of the products with Asp **10** and Glu **11** in the bicarbonate environment was characteristic by a strong release of the carbon dioxide reflecting in an unexpectedly high percentage of **1** (i.e., unreacted) and AA-monosubstituted compounds in the reaction mixtures. When comparing the bicarbonate and carbonate media, similar results were obtained for the product with Tyr 4. The synthesis of this derivative provided many unidentified impurities, as indicated by the HPLC-DAD and NMR analysis and percentual amounts in Table 1a.

*TEA synthetic procedure*. Very low yields of the desired products (below 10%) and high portions of the AA-monosubstituted derivatives were obtained with the polar uncharged (**6**–**9**) and negatively charged (**10**) AA. An exception was the conjugate with Glu **11** (yield ca. 20%). When comparing the yields of conjugates **3** (Phe, 2.5%) and **4** (Tyr, 0.2%), then **5** (Trp, 55%) and **2** (Ala, 45%), TEA was more useful for the synthesis of conjugates with the hydrophobic amino acids possessing an aliphatic side chain. In general, however, the TEA strategy does not represent an optimum solution for the preparation of 1,3,5-triazinyl-substituted benzenesulfonamide derivatives containing a symmetric pair of the amino acids as no benefit of the water based strategies was overcome.

*DMF and THF synthetic procedures*. The synthetic procedures with DMF or THF solvents brought even worse results than the TEA procedure, as it was demonstrated via Tyr and Trp derivatives. The reactants were not properly solubilized with these solvents and no or very low amount of the precipitate was formed (below 30%). The HPLC-DAD-MS analysis confirmed only the presence of impurities (mainly monosubst. OH- or disubst. AA + OH-precursor **1**, exceeding 30%, as well as big number of other unidentified impurities) in the reaction mixtures even after 24 h from the beginning of synthesis.

It can be summarized that 1,3,5-triazinyl-aminobenzenesulfonamide conjugates disubstituted with a symmetric pair of amino acids can be advantageously prepared in water environment containing sodium carbonate or sodium bicarbonate, depending on the type of implemented amino acid. The comprehensive study, based on the evaluation of different synthetic strategies via their corresponding HPLC-ESI-DAD/QTOF/MS analytical products profiles, revealed, the water medium with sodium carbonate was favorable for a majority of the derivatives with the proteinogenic amino acids possessing a hydrophobic side chain. On the other hand, the favorable conditions for a majority of the derivatives with the polar neutral amino acids were represented by the water medium with sodium bicarbonate. The derivatives with the amino acids possessing a negatively charged side chain could be preferably obtained in carbonate medium instead of bicarbonate. In comparison with the studied organic media, the water-based ones provided considerably higher product yields and purities (i.e., better selectivity towards the disubstitution products). The shorter synthetic times and environmentally friend (green) synthetic conditions are included among their additional benefits.

### 2.2. QSAR Study of Inhibition Activities of Sulfonamide Derivatives against hCA (I, II, IV, IX) Isoforms

Quantitative structure-activity relationship (QSAR) is used widely to screen and predict various forms of activity of molecules (e.g., inhibition). The general assumption for QSAR studies is that physical, chemical and biological properties of compounds strongly depend on their structure [44]. The procedure for establishing a reliable QSAR predictive model involves a number of important steps that are closely related: (a) the selection of representative compounds serving for the calibration and validation of QSAR (i.e., the training set and the validation set), (b) the multivariate characterization of these sets, (c) the biological profiling of these compounds, (d) the QSAR modeling, and (e) the validation of the resulting QSAR model [45]. The main reason for building up a QSAR model for a given problem is that it gives the information on how changes in the chemical structure of the compounds impact their biological activity. In this study, a QSAR model was proposed and applied for the prediction of inhibition activities of new sulfonamide derivatives (incorporating triazine with a symmetric pair of polar and some non-polar proteinogenic amino acids) against four isoforms of hCAs (hCA I, hCA II, hCA IV, and hCA IX).

#### 2.2.1. Data Set

The original data matrix comprised of molecular descriptors for 37 s-triazinyl-substituted aminoalkylbenzenesulfonamides altogether with 30 known experimental values for the inhibition constants (K_I_s) against hCA I, hCA II, hCA IV, and hCA IX isoforms taken from the previous work [43]. The data set was split into a training set (70%) and a validation set (30%) randomly. The training set of 21 derivatives was used to adjust the parameters of the models, and the validation set of 9 derivatives was applied for the evaluation of its prediction ability. The remaining 7 new derivatives with yet unknown inhibition values were later calculated by these prediction models.

Primary data processing included the data transformation, descriptive and robust statistical treatment. All variables were transformed by the min-max transformation to values within the interval of 0 to +1. Molecular descriptors (i.e., physical-chemical characteristics) without any relevant information (zero variability) were omitted from the data matrix. The final data matrix comprised of 37 objects (rows representing derivatives of sulfonamides) and 2414 molecular descriptors (table columns) along with 7 columns containing the inhibition values (hCA I, hCA II, hCA IV, hCA IX) and labeling information (Number, Code, and Name), altogether 106,216 entries.

#### 2.2.2. Molecular Optimization and Descriptor Calculation

The molecular structures of all 37 sulfonamide derivatives were drawn using ChemSketch software [46]. Then, the prepared structures were exported to Spartan ’14 [47] in order to build up and optimize their 3D structures. The effect of solvent as well as protonation was taken into account during the modelling process. In a first step, a “Conformer distribution” calculation using Monte-Carlo algorithm was run for 10,000 conformers. In this case, the molecular mechanics force field in water (MMFFaq) was utilized to establish an equilibrium conformation. The 100 conformers with the lowest energy generated by MMFFaq were saved and then “Equilibrium geometry” optimization was subjected to a semi-empirical quantum method PM3. After the 100 conformers have been PM3 optimized, the conformer with the lowest energy was exported in mol2 file for further calculation of all molecular descriptors by Dragon 6 software [48].

#### 2.2.3. QSAR Modelling

The pretreated data matrix provided by the Dragon software contained 2414 molecular descriptors from various blocks, such as constitutional, topological, charge descriptors, 2D autocorrelations, etc. It is obvious, that all these descriptors could not be used for QSAR studies from practical point of view. Therefore, one of the most important tasks, prior to modeling, was the selection of relevant descriptors providing maximum information about the derivatives. Selection of the most important variables was calculated using “Data Mining Feature Selection” tool by Statistica 12 software package [49]. The importance/relevance of molecular descriptors with their relation to inhibition against the hCAs was expressed by a probability value (*p*), and thirty most significant descriptors were kept for the subsequent QSAR modeling via artificial neural networks. The list of selected descriptors is shown in Table S1, and the corresponding discussion to the selection of molecular descriptors is in the Section S11 of the Supplementary Material.

#### 2.2.4. Application of Artificial Neural Networks

Each set of the selected molecular descriptors for the prediction of inhibitory activity against the particular hCAs has been used in the training of non-linear predictive models, specifically, artificial neural networks (ANN) with three-layer-perceptron (3-MLP) architecture and backpropagation (BP) learning algorithms. Thirty derivatives of sulfonamides with known values of inhibitory activity have been used in the ANN training process, in which 21 randomly selected derivative samples (70%) formed a training set and 9 samples served for a validation purpose (30%). The remaining thirty sulfonamides, with inhibition values not experimentally measured yet, were used as a testing set and their values were predicted by the ANN models.

A multitude of networks with different architectures were examined during the training procedure. A variety of algorithms for different neural network types were automatically tested and the best alternatives were selected. The number of neurons in the input layer was set by the number of molecular descriptors (30), the output layer with one neuron represented calculated inhibition value. The optimal choice of hidden neurons was found by examining several types of the three-layer-perceptron (3-MLP) with regard to the corresponding final root mean squared error (RMSE). The learning process was initialized by the Broyden–Fletcher–Goldfarb–Shanno (BFGS) training algorithm with the number of cycles set to 200. Weight decay value was set to 10^−6^ for the hidden layer and 10^−5^ for the output layer.

In order to evaluate the accuracy of the network, and, furthermore, the performance of the ANN model, RMSE has been utilized. The RMSE refers, how concentrated the data is around the line of best fit. It can be stated that the model has optimal accuracy if the RMSE value reaches zero. Regression coefficient was another statistical indicator for rating the goodness of fit. It shows how much two groups of data are correlated. If its value approaches to 1, that means the performance of the model is the best.

The best results for hCA I calculations were achieved with the neural network architecture MLP 30-17-1 using a hyperbolic tangent function (tanh) for the hidden neurons activation and an exponential one (exp) for the output neuron. The training performance (regression coefficient) reached the value of 0.9643 with the error (RMSE) 0.0039 and the validation performance was 0.9411 with the validation error 0.0112. In case of the neural network for hCA II prediction, MLP 30-13-1 with a hyperbolic tangent activation function for the hidden neurons and with a logistic function (log) for the output neuron activation was the network with the best prediction performance. The training performance was 0.9972 with the error 0.0003 while the validation performance was 0.9531 with the error 0.0064. The network architecture MLP 30-19-1 with the same activation functions as in previous case (tanh, log) was found to be the most suitable for the prediction of hCA IV inhibition data, in which the performance of training set reached the value of 0.9994 (error 0.0001) and validation 0.9441 (error 0.0098). MLP 30-12-1 (tanh, log) for the hCA IX calculations provided the following results: performance of training 0.9927 (error 0.0011), validation performance 0.9748 (error 0.0066). Summarized information about all calculated artificial neural networks is listed in Table 2.

#### 2.2.5. Evaluation of Predicted Inhibition Activities of New Sulfonamide Derivatives

The aim of the QSAR modeling was to develop a model of the inhibitory activity of sulfonamide derivatives against various hCAs, to better understand the structural features of these compounds. The optimum model for given data set should help to design new analogues with better desired properties in the future. In this work, the optimum models were applied for the prediction of K_I_s of the new derivatives, 1,3,5-triazinyl-substituted benzenesulfonamides incorporating a symmetric pair of the proteinogenic amino acids possessing a hydrophobic side chain (Ala, Tyr, Trp) or a polar uncharged chain (Gln, Asn, Ser, Thr), against hCA I, II, IV, IX isoforms. The experimental [43] and recalculated (this work) inhibition constants (K_I_, nM) of the 30 published [43] sulfonamide inhibitors against the physiologically relevant hCA isoforms, such as hCA I, II, IV, and tumor-associated hCA IX, are listed in Table 3.

The comparison of the predicted values of hCA I inhibition with the experimentally acquired results showed a relatively good match, with an exception of the values for derivatives SA Gly (4550.0 vs. 277.0 nM, used as a sample in training set), SAM Leu (96.9 vs. 387.6 nM, used in validation set), SA Met (346.7 vs. 798.1 nM, training set), SAM Phe (67.1 vs. 364.1 nM, training set), and SAE Pro (87.0 vs. 188.4 nM, validation set).

When inspecting the inhibitory values for hCA II, it can be clearly said that the very good agreement between the experimental and predicted values have been achieved. In this case, the distribution of errors was slightly higher among the derivatives with lower inhibitory values than among those with the higher K_I_s.

The predicted values of hCA IV inhibition showed significantly different values in case of several derivatives, namely SAM Gly (867.3 vs. 1651.2 nM, used as a part of validation set), SAM Ile (76.3 vs. 154.9 nM, used in training set), SAE Met (2336.1 vs. 60.1 nM, validation set), SAE Phe (374.5 vs. 47.5 nM, training set), and SAE Pro (2206.9 vs. 719.9 nM, training set). The rest of the derivatives were in a good match with the experimental results.

The comparison of the experimental and predicted values of sulfonamides against the tumor-associated hCA IX showed mixed results. While the prediction of several derivatives provided comparable results with the experiment, the others were significantly different. The biggest difference has been observed in case of SAE B-Ala (134.2 vs. 8.5 nM, used in training set), SAM Glu (193.2 vs. 19.5 nM, used in validation set), SAM Gly (145.8 vs. 10.5 nM, validation set), SAE Gly (330.4 vs. 8.4 nM, training set), SA Pro (295.0 vs. 56.1 nM, validation set), and SAM Val (1371.8 vs. 457.2 nM, validation set). Although the RMSE as well as the regression coefficient for both the training and validation set indicated a well-trained ANN model, the recalculated inhibitory values (after the re-transformation from min-maxed range <0, +1> to the original scale) were affected by higher errors in comparison with the hCA I, hCA II, or hCA IV models. It can be logically supposed that an enhancement of the prediction would be achieved for larger datasets (continually produced in our subsequent works aimed at the triazinyl-aminobenzenesulfonamide derivatives with amino acids).

The predicted inhibitory constants for 7 new sulfonamide derivatives with unknown inhibition activity against the four hCA isoforms, obtained by the most suitable ANN models, are listed in Table 4. The predicted values of new compounds showed only weak activity as inhibitors of hCA I (K_I_s ≥ 239.6 nM) and hCA II (K_I_s ≥ 235.8 nM). The predicted inhibitory activity towards hCA IV was negligible (K_I_s ≥ 2328.9 nM). On the other hand, a lot of compounds showed strong activity against hCA IX isoform (those with K_I_s in the range of 8.4–29.6 nM). Among them were three derivatives with polar amino acids (Gln, Ser, Thr) and one derivative with non-polar amino acid (Ala). The rest of derivatives exhibited weak activity as inhibitors of hCA IX (K_I_s ≥ 192.9 nM). Hence, the substitution with polar amino acids seems to be a promising way to create the sulfonamide derivatives with a strong activity against hCA IX isoform; for the presented new polar derivatives it is comparable or even stronger than this one of acetazolamide. Generally, benzenesulfonamide derivatives represent an attractive group of hCA IX inhibitors that can act even in picomolar levels, namely 1,3,5-triazinylbenzenesulfonamide disubstituted with ethoxy moieties, K_I_ 120 pM [32], 1,3,5-triazinylaminomethylbenzenesulfonamide monosubstituted with 4-aminophenol, K_I_ 400 pM [40], 4,4′-disulfanediyldibenzenesulfonamide, K_I_ 500 pM [50], cyclohexyl-substituted thiobenzenesulfonamide, K_I_ 600 pM [50], tetrahydroquinazolinyl-aminobenzenesulfonamides, K_I_ 550–890 pM [51], 1,3,4-thiadiazole-containing cyanoacrylamidebenzensulfonamide, K_I_ 330 pM [52], 3/4-(3-aryl-3-oxopropenyl)aminobenzenesulfonamides with the lowest K_I_s ranging in the interval of 210–820 pM [53], and pyridazinone-containing benzenesulfonamides [30] or 1,2,4-oxadiazole-containing benzenesulfonamides [54] with the lowest K_I_s ranging in the intervals of 240–700 or 89–880 pM, respectively. From among the new compounds studied in our work, the polar sulfonamide derivative, disubstituted with Gln, exhibited the highest predicted selectivity (139.1) towards the tumor-associated, membrane-bound hCA IX isoform, expressed via the K_I_ ratio of hCA II/hCA IX (see Table 4).

### 2.3. Molecular Modeling

**Figure 2:**
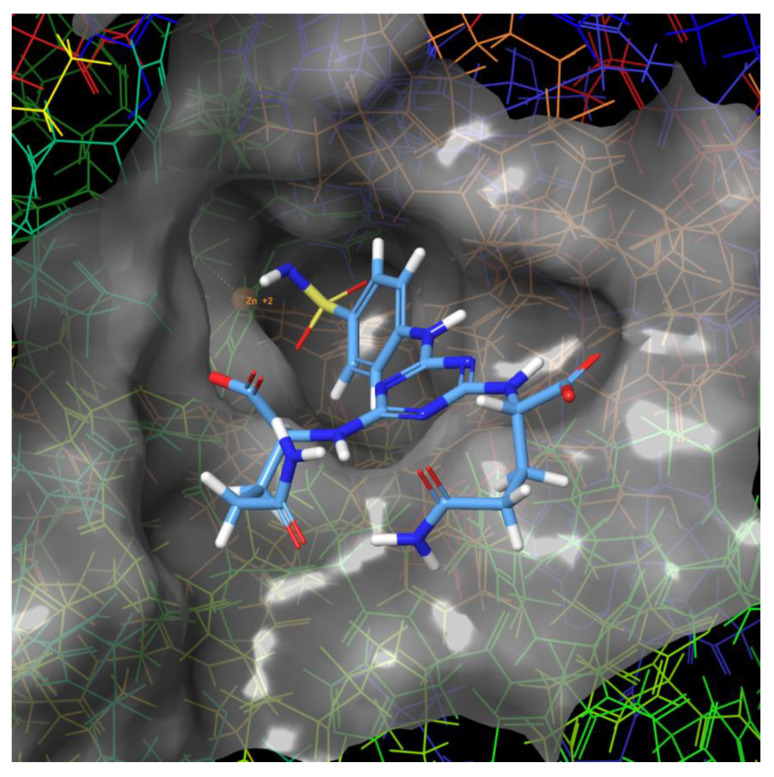
Molecular surface of the binding site of hCA II with docked compound **7** (with Gln).

The structures of molecular complexes including hCA II or hCA IX with the new derivative (Gln) exhibiting the highest predicted selectivity (hCA II/hCA IX) were visualized using Maestro Software [55]. Molecular surface of the binding site of hCA II with docked ligand **7** (with Gln) is shown on Figure 2.

Ligands were prepared in LigPrep package [56] with respect to their geometry (geometry optimization by OPLS3, generation of all optical isomers, low-energy ring conformations) and protonation states (according to residue pKa’s), and the most convenient structure was docked into the hCAII or hCAIX isoenzyme. Protein set up of hCAII and hCA IX implied the import and processing of the protein chain (i.e., removing of water, hets, fixing of missing chains) with the aid of Protein Preparation Wizard [57] using the 2.2 Å resolution, where PDB entry 3K34 was applied as a basis for hCAII and PDB entry 5FL4 for hCAIX build up. The preparation was done with water molecules within 5 Å of the ligand initially retained, but deleted for subsequent docking. Bond orders were assigned and hydrogen atoms added, with protonation states for basic and acidic residues based on residue pKa’s at normal pH (7.0). Sulfonamide group was N-deprotonated as it is well known that in this form it serves as zinc binding site. Complex of docked ligand onto protein was minimized by MacroModel [58] using OPLS3e force field, where constrains were applied to the backbone of the protein.

**Figure 3:**
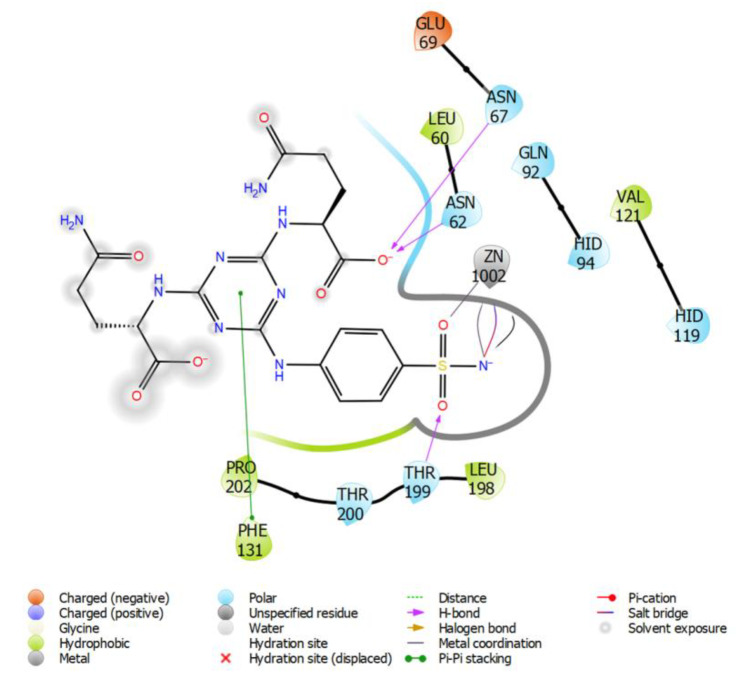
Intermolecular interactions of compound **7** (with Gln) docked into hCA II.

Intermolecular interactions of compound **7** (with Gln) docked into human isozyme II are depicted in Figure 3. The threonine 199 creates H-bond with the sulfonamide functional group and the asparagines 62 and 67 are attached to carboxylic group of Gln residue of the ligand. Triazine cycle forms a π-π interaction with the phenylalanine 131. These interactions can be compared with the already known crystal structures such as 3MNA and 3MMF. In case of 3MNA, Thr199 creates H-bonds with the sulfonamide functional group as well as Gln92 with the triazine cycle. Intermolecular interactions in 3MMF involve the H-bonds of Thr199 with the sulfonamide residue of the ligand as well as Gln92 with the triazine cycle. Similarly, the π-π interaction of triazine circle with the Phe131 can be also observed.

**Figure 4:**
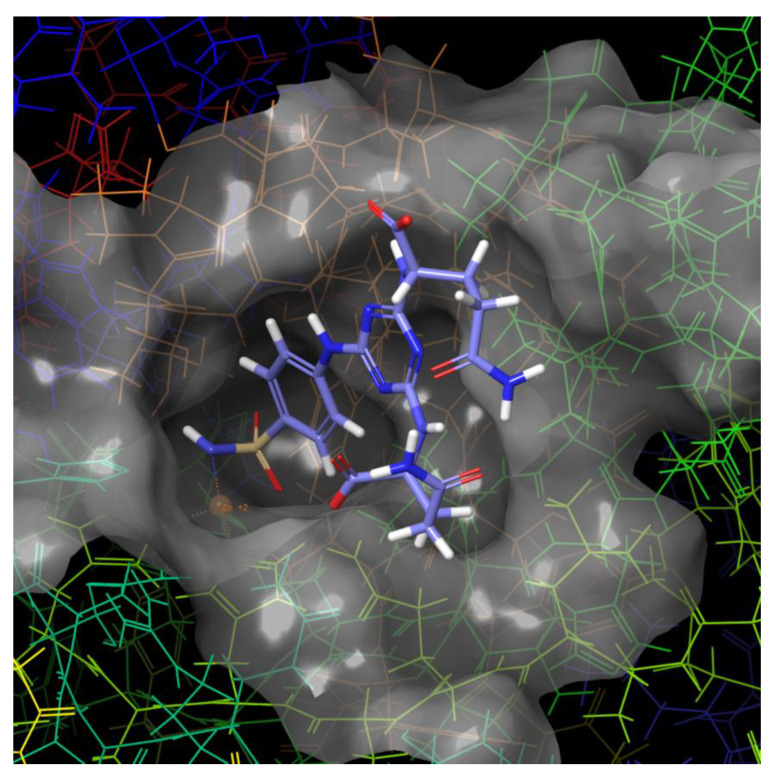
Molecular surface of the binding site of hCA IX with docked compound **7** (with Gln).

**Figure 5:**
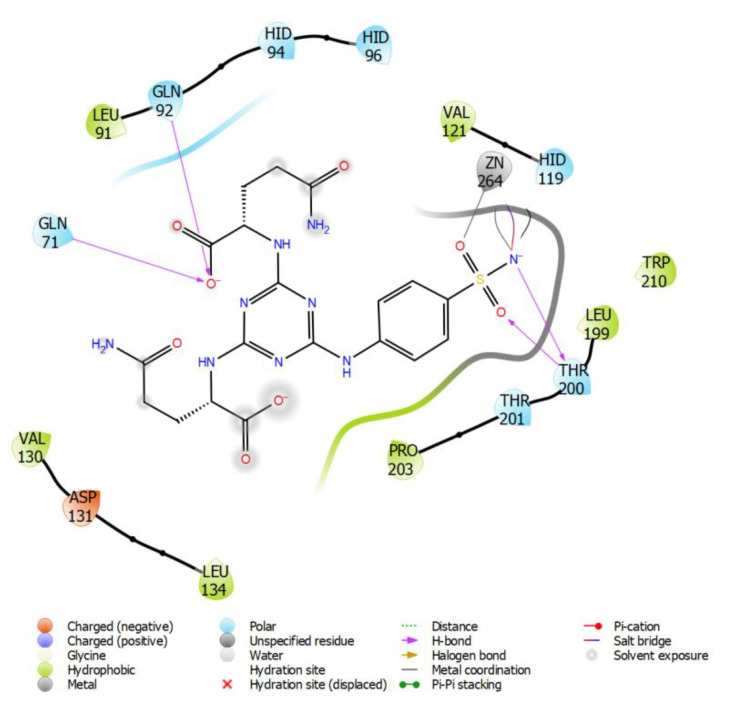
Intermolecular interactions of compound **7** (with Gln) docked into hCA IX.

The pose of molecule **7** (with Gln) in hCA IX is slightly different from the pose in hCA II (Figure 4 and Figure 5). The threonine 200 forms proton-donor and proton-acceptor H-bonds with the sulfonamide group of the ligand, and the carboxylic group of Gln residue attached to the triazine cycle forms H-bonds with the glutamines 71 and 92.

## 3. Materials and Methods

### 3.1. Materials and Instruments for the Synthesis and Analysis

All solvents and reagents were purchased from commercial suppliers (AppliChem GmbH, Darmstadt, Germany; Sigma Aldrich, St. Louis, MO, USA and VWR International, Vienna, Austria) and used without further purification as supplied.

All the reactions were monitored by the thin-layer chromatography (TLC) performed on the Silica gel plates 60 F254 (Merck, Darmstadt, Germany) with the UV visualization (245 nm) and hexane/ethyl acetate or dichloromethane/methanol as eluents.

The crude products were purified by a semi-preparative LC on a Shimadzu instrument containing two quarternary Prep-LC pumps LC-20AP, autosampler for preparative injection SIL-10AP, column oven CTO-20A, PDA detector SPD-M20A, and fraction collector FRC-10A (Shimadzu, Kyoto, Japan). The preparative LC was performed employing either a RP-C18 column (Kromasil C18, 10 µm, 250 × 10 mm, Nouryon, Bohus, Sweden) or an HILIC column (Kromasil 60-10 Sil, 10 µm, 250 × 10 mm, Nouryon, Bohus, Sweden) with a mixture of 50 mM/100 mM solution of ammonium bicarbonate (mobile phase A, NH_4_HCO_3_) and methanol/acetonitrile (mobile phase B) in isocratic conditions optimized individually for each sulphonamide derivative (**2–11**). The flow rate of mobile phase was set at 15 mL/min. The detailed chromatographic conditions used for the purification by the semi-preparative LC are summarized in Table 5. The main chromatographically prepared fractions, containing a desired product, were lyophilized for 24 h with the collector temperature set at −84 °C and pressure 0.003 mbar using FreeZone 2.5 Liter Benchtop system (Labconco Corporation, Kansas City, MO, USA). Desired solid products with high purity (>98%) were obtained in this way (for the purity data see the Section 3.2.2. Pure Products Characterization).

The nuclear magnetic resonance (^1^H NMR, ^13^C NMR) spectra were measured in DMSO-*d_6_* as a solvent on an Agilent MR400 (400 MHz) spectrometer. All NMR spectra were referenced to tetramethylsilane (TMS, δ = 0.00 ppm) as an internal standard and reported as follows: chemical shift in ppm, multiplicity (b = broad, s = singlet, d = doublet, t = triplet, q = quartet, dd = doublet of doublet, m = multiplet). The proton exchange is observed for all products, when ^1^H NMR spectra showed characteristic broad singlets that correspond to NH and OH proton exchange.

Infrared (IR) spectra (in KBr plates) were recorded on a Perkin-Elmer UATR Two (PerkinElmer Ltd., Beaconsfield, UK) spectrometer. All IR spectra were presented in wavenumbers (υ_max,_ cm^−1^) and signal intensities were denoted as follows: br = broad, w = weak, m = medium, s = strong.

All of the LC-MS/DAD data were obtained on an LC Agilent Infinity System (Agilent Technologies, Santa Clara, CA, USA) equipped with a gradient pump (1290 Bin Pump), an automatic injector (1260 HiPals), and a column thermostat (1290 TCC). The LC system was coupled with a photodiode array detector (Infinity 1290 DAD) and a quadrupole time-of-flight mass spectrometer (6520 Accurate Mass Q-TOF LC/MS). Q-TOF was equipped with an electrospray ionization source operated in positive ionization mode. All measurements were performed with the following MS parameters: drying gas temperature 360 °C, drying gas flow 12 L·min^−1^, nebulizing gas pressure 60 psi, ESI source voltage 3500 V, fragmentor voltage 100 V, collision gas N_2_. The mass spectrometer was tuned using external calibration before the analysis. Considering the HPLC-DAD analysis (for the purpose of purity evaluation), the peak areas of the compounds and respective impurities at wavelength of 254 nm were measured.

The HPLC analyses were performed using SeQuant^®^ ZIC^®^-HILIC column, 2.1 × 100 mm, 3.5 μm (Merck KGaA, Darmstadt, Germany) as a stationary phase and a 10 mM ammonium acetate aqueous solution with an addition of 0.1% acetic acid (*v/v*) (A) and 100% acetonitrile (B) as mobile phases. The elution was performed in a gradient mode using the following composition of the mobile phases: 0.0 min–95% B; 0.0–20.0 min–linear change to 40% B; 20.0–25.0 min–40% B. The column was re-equilibrated with the initial composition of the mobile phases for 6 min. The flow rate was 0.500 mL/min and the column was kept at a temperature of 40 °C.

For each sample, a 1.0 mg of the solid product was dissolved in 1.00 mL of 80% acetonitrile solution with an addition of 5 mM ammonium bicarbonate. The dissolved samples were filtered through a 0.22 μm nylon syringe filter and a 1.0 μL volume was used for the HPLC-MS/DAD analysis.

### 3.2. Synthetic Procedures and Products Characterization

The developed synthetic procedures were applied for the preparation of compounds **2–11**, for the general methods description see Section 3.2.1. and for the products characterization see Section 3.2.2. To obtain the highest possible purity of the demanded products (>98%), a semi-preparative chromatography method was developed (for the method instrumentation and conditions see Section 3.1.) and applied for the crude products **2–11**. This purification approach was essential especially for several problematic derivatives, namely those with Asn, Gln, and Tyr (their purity was below 50%). The measured HPLC-DAD-MS, NMR, and IR spectra of the products 2–11 are in the Supplementary Material, Sections S1–S10.

#### 3.2.1. General Methods for the Synthesis of Disubstituted Derivatives of 4-(4′,6′-dichloro-1′,3′,5′-triazine-2′-ylamino)benzenesulfonamide **2**–**11**

The precursor 4-(4′,6′-dichloro-1′,3′,5′-triazine-2′-ylamino)benzenesulfonamide **1** was prepared according to the methodology published previously [35]. A reaction mixture contained the precursor **1** (0.1 g, 1 equiv.) and amino acid (3 equiv.). In the water based procedures (carbonate and bicarbonate) the reaction mixture was stirred in H_2_O (2 mL) at the room temperature for 10 min. The aqueous solution of either Na_2_CO_3_ or NaHCO_3_ (4 equiv./5 equiv. for dicarboxylic AA) was added dropwise into the reaction mixture. Then the mixture was refluxed for 20–24 h (in case of Na_2_CO_3_ procedure) or 8–12 h (in case of NaHCO_3_ procedure) until the completion of reaction was confirmed by TLC. Compounds were precipitated by 1M HCl or 1M NaOH addition (up/down to proper pH depending on the amino acid) until a maximum amount of the precipitate was produced.

In the organic solvent based procedures (TEA, THF, DMF) a mixture of **1** (0.1 g, 1 equiv.) and particular anhydrous organic (10 mL of THF with 2 equiv. of Na_2_CO_3_ acting under an argon atmosphere, or 10 mL of DMF with 4 equiv. of a catalyzing agent, either NaHCO_3_ or KF) or water-organic (0.2% *v/v* TEA in water) solvent was stirred at the room temperature for 30 min. An amino acid (3 equiv.) was dissolved in 0.2% *v/v* TEA or THF or DMF and added dropwise into the mixture. The reaction mixture with TEA was stirred at 90 °C for 5 h while the THF and DMF mixtures were stirred and refluxed overnight until the completion of reaction was confirmed by TLC. The reaction mixtures were cooled down to enhance the precipitation of products, and neutralized by 1M HCl (in case of TEA procedure).

The resulting precipitates were isolated by filtration, washed with ethanol (when using water based media), and dried under high vacuum before analysis or purification by the preparative LC.

#### 3.2.2. Pure Products Characterization

2,2′-((6-([4-Sulfamoylphenyl]amino)-1,3,5-triazine-2,4-diyl)bis(amino))dipropanoic acid **2**

White solid; yield 92%. HILIC-DAD purity 99.56%. IR (KBr, υ_max_ cm^−1^): 3249 (br w, NH), 2972 (w), 1715 (w, C=O), 1630 (w, C=C), 1557 (s), 1502 (s), 1406 (s) 1316 (m, SO_2_NH_2_), 1243 (w, C-N), 1154 (s, SO_2_NH_2_), 1099 (m). ^1^H NMR (400 MHz, DMSO-*d_6_*, 90 °C) δ: 9.03 (br s, 1H, Ar-**NH**), 7.90 (d, 2H, *J* = 8.8 Hz, 2 × Ar-H_a_), 7.67 (d, 2H, *J* = 8.8 Hz, 2 × Ar-H_b_), 6.56 (br s, 2H, 2 × **NH**-CH), 4.34 (q, 2H, *J* = 7.2 Hz, 2 × H-C(2)), 1.36 (d, 6H, *J* = 7.0 Hz, 2 × H-C(3)). ^13^C NMR (100.58 MHz, DMSO-*d_6_*, 90 °C) δ: 175.06, 165.65, 164.56, 144.22, 136.75, 126.68, 119.18, 50.01, 18.54. HRMS (ESI/QTOF, *m/z*): [M + H]^+^ Calcd. for [C_15_H_19_N_7_SO_6_H]^+^ 426.1190; Found: 426.1196.

2,2′-((6-([4-Sulfamoylphenyl]amino)-1,3,5-triazine-2,4-diyl)bis(amino))di(3-phenylpropanoic acid) **3**

White solid; yield 91%. HILIC-DAD purity 98.74%. IR (KBr, υ_max_ cm^−1^): 3267 (br w, NH), 3028 (w), 1716 (w, C=O), 1654 (w, C=C), 1557 (s), 1496 (s), 1403 (s) 1321 (m, SO_2_NH_2_), 1241 (w, C-N), 1153 (s, SO_2_NH_2_), 1098 (w). ^1^H-NMR (400 MHz, DMSO- *d_6_*, 90 °C) δ: 9.10 (br s, 1H, Ar-**NH**), 7.84 (d, 2H, *J* = 8.9 Hz, 2 × Ar-H_a_), 7.66 (d, 2H, *J* = 8.8 Hz, 2 x Ar-H_b_), 7.23 (s, 2H, SO_2_NH_2_), 7.22−7.19 (m, 8H, 8 x Ar-H), 7.16–7.12 (m, 2H, 2 × Ar-H), 6.45 (br s, 2H, 2 × **NH**-CH), 4.61 (br s, 2H, 2 × H-C(2)), 3.18 (dd, 2H, *J* = 13.9 Hz, *J* = 5.2 Hz, 2 × H_a_-C(3)), 3.06 (dd, 2H, *J* = 13.9 Hz, *J* = 7.8 Hz, 2 × H_b_-C(3)). ^13^C NMR (100.58 MHz, DMSO-*d_6_*, 90 °C) δ: 173.70, 165.80, 164.43, 144.07, 138.79, 136.84, 129.62, 128.37, 126.58, 119.22, 55.57, 37.70. HRMS (ESI/QTOF, *m/z*): [M + H]^+^ Calcd. for [C_27_H_27_N_7_SO_6_H]^+^ 578.1816; Found: 578.1829.

2,2′-((6-([4-Sulfamoylphenyl]amino)-1,3,5-triazine-2,4-diyl)bis(amino))di(3-(4-hydroxyphenyl)propanoic acid) **4**

White solid; yield 29%. HILIC-DAD purity 98.24%. IR (KBr, υ_max_ cm^−1^): 3228 (br w, NH), 3028 (w), 1715 (w, C=O), 1558 (s), 1513 (s), 1405 (s) 1321 (m, SO_2_NH_2_), 1240 (w, C-N), 1154 (s, SO_2_NH_2_), 1099 (w). ^1^H NMR (400 MHz, DMSO-*d_6_*, 90 °C) δ: 8.99 (br s, 1H, Ar-**NH**), 7.85 (d, 2H, *J =* 8.9 Hz, 2 × Ar-H_a_), 7.67 (d, 2H, *J =* 8.8 Hz, 2 x Ar-H_b_), 7.00 (d, 4H, *J =* 8.3 Hz, 4 × Ar-H), 6.63 (d, 4H, *J =* 8.3 Hz, 4 × Ar-H), 6.30 (br s, 2H, 2 × **NH**-CH), 5.25 (br s, 2H, SO_2_NH_2_), 4.53–4.48 (m, 2H, 2 × H-C(2)), 3.06 (dd, 2H, *J* = 13.8 Hz, *J* = 5.2 Hz, H_a_-C(3)), 2.95 (dd, 2H, *J* = 13.8 Hz, *J* = 7.2 Hz, H_b_-C(3)). ^13^C-NMR (100.58 MHz, DMSO-*d_6_*, 90 °C) δ ppm: 173.89, 165.74, 164.45, 156.22, 144.15, 136.75, 130.49, 128.84, 126.69, 119.18, 115.46, 55.91, 36.93. HRMS (ESI/QTOF, *m/z*): [M + H]^+^ Calcd. for [C_27_H_27_N_7_SO_8_H]^+^ 610.1715; Found: 610.1693.

2,2′-((6-([4-Sulfamoylphenyl]amino)-1,3,5-triazine-2,4-diyl)bis(amino))di-(3-(1*H*-indol-3-yl)propanoic acid) **5**

White solid; yield 87%. HILIC-DAD purity 98.35%. IR (KBr, υ_max_ cm^−1^): 3402 (w, NH), 3272 (br w, NH), 2966 (w), 1715 (w, C=O), 1559 (s), 1502 (s), 1408 (s), 1326 (m, SO_2_NH_2_), 1234 (w, C-N), 1154 (s, SO_2_NH_2_), 1097 (w). ^1^H NMR (400 MHz, DMSO-*d_6_*, 90 °C) δ: 10.54 (s, 2H, 2 x H-N_ind_), 9.00 (br s, 1H, Ar-**NH**), 7.84 (d, 2H, *J* = 8.9 Hz, 2 x Ar-H_a_), 7.65 (d, 2H, *J* = 8.8 Hz, 2 x Ar-H_b_), 7.53 (d, 2H, *J* = 7.9 Hz, 2 × Ar_ind_-H), 7.30 (d, 2H, *J* = 8.1 Hz, 2 x Ar_ind_-H), 7.12 (s, 2H, 2 x Ar_ind_-H), 7.04 - 7.00 (m, 2H, 2 x Ar_ind_-H), 6.95 - 6.91 (m, 2H, 2 x Ar_ind_-H), 6.33 (br s, 2H, 2x **NH**-CH), 5.21 (br s, 2H, SO_2_NH_2_), 4.69 (br s, 2H, 2 x H-C(2)), 3.31 (dd, 2H, *J* = 14.6 Hz, *J* = 4.9 Hz, H_a_-C(3)), 3.19 (dd, 2H, *J* = 14.4 Hz, *J* = 7.3 Hz, H_b_-C(3)). ^13^C NMR (100.58 MHz, DMSO-*d_6_*, 90 °C) δ: 174.21, 165.86, 164.46, 144.09, 136.72, 128.19, 126.69, 124.01, 121.19, 119.16, 118.74, 111.70, 110.93, 55.08, 27.80. HRMS (ESI/QTOF, *m/z*): [M + H]^+^ Calcd. for [C_31_H_29_N_9_SO_6_H]^+^ 656.2034; Found: 656.2051.

2,2′-((6-([4-Sulfamoylphenyl]amino)-1,3,5-triazine-2,4-diyl)bis(amino))di(4-amino-4-oxobutanoic acid) **6**

White solid; yield 56%. HILIC-DAD purity 98.11%. IR (KBr, υ_max_ cm^−1^): 3195 (br m, NH), 2972 (w), 1718 (w, C=O), 1659 (m, C=C), 1558 (s), 1496 (s), 1404 (s), 1315 (m, SO_2_NH_2_), 1239 (w, C-N), 1152 (s, SO_2_NH_2_), 1098 (w). ^1^H NMR (400 MHz, DMSO-*d_6_*, 90 °C) δ: 8.99 (br s, 1H, Ar-**NH**), 7.88 (d, 2H, *J* = 8.8 Hz, 2 × Ar-H_a_), 7.68 (d, 2H, *J* = 8.8 Hz, 2 x Ar-H_b_), 7.18 (br s, 2H, SO_2_NH_2_), 6.53 (br s, 2H, 2 × **NH**-CH), 4.61 (br s, 2H, 2 × H-C(2)), 2.62 (d, 4H, *J* = 5.8 Hz, 2 × H-C(3)). ^13^C NMR (100.58 MHz, DMSO-*d_6_*, 90 °C) δ: 173.68, 172.61, 165.73, 164.53, 144.11, 136.81, 126.76, 119.25, 51.60, 38.58. HRMS (ESI/QTOF, *m/z*): [M + H]^+^ Calcd. for [C_17_H_21_N_9_SO_8_H]^+^ 512.1316; Found: 512.1299.

2,2′-((6-([4-Sulfamoylphenyl]amino)-1,3,5-triazine-2,4-diyl)bis(amino))di(5-amino-5-oxopentanoic acid) **7**

White solid; yield 28%. HILIC-DAD purity 98.01%. IR (KBr, υ_max_ cm^-1^): 3204 (br w, NH), 2968 (w), 1721 (w, C=O), 1660 (m, C=C), 1576 (s), 1507 (s), 1408 (s), 1321 (m, SO_2_NH_2_), 1240 (w, C-N), 1154 (s, SO_2_NH_2_), 1099 (w). ^1^H NMR (400 MHz, DMSO-*d_6_*, 90 °C) δ: 8.98 (br s, 1H, Ar-NH), 7.89 (d, 2H, *J* = 8.8 Hz, 2 × Ar-H_a_), 7.67 (d, 2H, *J* = 8.8 Hz, 2 × Ar-H_b_), 6.98 (br s, 2H, SO_2_NH_2_), 6.54 (br s, 2H, 2 × **NH**-CH), 4.33-4.29 (m, 2H, 2 x H-C(2)), 2.22 - 2.18 (m, 4H, 2 × H-C(4)), 2.10–2.01 (m, 2H, H_a_-C(3)), 1.97–1.88 (m, 2H, H_b_-C(3)). ^13^C-NMR (100.58 MHz, DMSO-*d_6_*, 90 °C) δ: 174.61, 174.21, 165.92, 164.52, 144.20, 136.72, 126.71, 119.19, 54.42, 32.28, 28.19. HRMS (ESI/QTOF, *m/z*): [M + H]^+^ Calcd. for [C_19_H_25_N_9_SO_8_H]^+^ 540.1634; Found: 540.1615.

2,2′-((6-([4-Sulfamoylphenyl]amino)-1,3,5-triazine-2,4-diyl)bis(amino))di(3-hydroxypropanoic acid) **8**

Light beige solid; yield 71%. HILIC-DAD purity 98.06%. IR (KBr, υ_max_ cm^−1^): 3210 (br w, NH), 2967 (w), 1711 (w, C=O), 1558 (s), 1497 (s), 1404 (s), 1308 (m, SO_2_NH_2_), 1241 (w, C-N), 1151 (s, SO_2_NH_2_), 1097 (w). ^1^H NMR (400 MHz, DMSO-*d_6_*, 90 °C) δ: 9.03 (br s, 1H, Ar-**NH**), 7.89 (d, 2H, *J* = 8.9 Hz, 2 × Ar-H_a_), 7.68 (d, 2H, *J* = 8.8 Hz, 2 × Ar-H_b_), 6.32 (br s, 2H, 2 × **NH**-CH), 4.28 - 4.25 (m, 2H, 2 × H-C(2)), 3.80 (dd, 2H, *J* = 10.3 Hz, *J* = 5.1 Hz, 2 x H_a_-C(3)), 3.63 (dd, 2H, *J* = 10.3 Hz, *J* = 5.4 Hz, H_b_-C(3)). ^13^C NMR (100.58 MHz, DMSO-*d_6_*, 90 °C) δ: 173.00, 165.94, 164.57, 144.16, 136.85, 126.71, 119.23, 62.81, 56.45. HRMS (ESI/QTOF, *m/z*): [M + H]^+^ Calcd. for [C_15_H_19_N_7_SO_8_H]^+^ 458.1089; Found: 458.1095.

2,2′-((6-([4-Sulfamoylphenyl]amino)-1,3,5-triazine-2,4-diyl)bis(amino))di(3-hydroxybutanoic acid) **9**

White solid; 87%. HILIC-DAD purity 99.31%. IR (KBr, υ_max_ cm^-1^): 3225 (br w, NH), 2968 (w), 1711 (w, C=O), 1559 (s), 1505 (s), 1400 (s), 1321 (m, SO_2_NH_2_), 1243 (w, C-N) 1153 (s, SO_2_NH_2_), 1098 (w). ^1^H NMR (400 MHz, DMSO-*d_6_*, 90 °C) δ: 9.04 (br s, 1H, Ar-**NH**), 7.89 (d, 2H, *J* = 8.9 Hz, 2 × Ar-H_a_), 7.68 (d, 2H, *J* = 8.8 Hz, 2 × Ar-H_b_), 6.14 (br s, 2H, 2 × **NH**-CH), 4.28 (br s, 2H, 2 x H-C(2)), 4.18–4.13 (m, 2H, 2 × H-C(3)), 1.09 (d, 6H, *J* = 6.3 Hz, 2 × H-C(4)). ^13^C NMR (100.58 MHz, DMSO-*d_6_*, 90 °C) δ: 172.95, 166.27, 164.62, 144.12, 136.87, 126.73, 119.22, 66.90, 59.54, 20.41. HRMS (ESI/QTOF, *m/z*): [M + H]^+^ Calcd. for [C_17_H_23_N_7_SO_8_H]^+^ 486.1402; Found: 486.1411.

2,2′-((6-([4-Sulfamoylphenyl]amino)-1,3,5-triazine-2,4-diyl)bis(amino))disuccinic acid **10**

White solid; yield 84%. HILIC-DAD purity 98.37%. IR (KBr, υ_max_ cm^−1^): 3202 (br w, NH), 2967 (w), 1716 (w, C=O), 1652 (w, C=C), 1557 (s), 1496 (s), 1398 (s), 1307 (m, SO_2_NH_2_), 1236 (w, C-N), 1150 (s, SO_2_NH_2_), 1064 (m). ^1^H-NMR (400 MHz, DMSO-*d_6_*, 90 °C) δ: 9.08 (s, 1H, Ar-**NH**), 7.88 (d, 2H, *J =* 8.8 Hz, 2 × Ar-H_a_), 7.68 (d, 2H, *J =* 8.9 Hz, 2 × Ar-H_b_), 6.30 (br s, 4H, SO_2_NH_2_, 2 × **NH**-CH), 4.40 (d, 2H, *J =* 7.4 Hz, 2 x H-C(2)), 2.65 (dd, 2H, *J =* 15.7 Hz, *J =* 3.1 Hz, 2 x H_a_-C(3)), 2.57 (dd, 2H, *J =* 15.7 Hz, *J =* 9.9 Hz, 2 × H_b_-C(3)). ^13^C-NMR (100.58 MHz, DMSO-*d_6_,* 90 °C) δ: 173.44, 172.41, 165.77, 164.55, 144.00, 136.95, 126.80, 119.29, 50.58, 38.24. HRMS (ESI/QTOF, *m/z*): [M + H]^+^ Calcd. for [C_17_H_19_N_7_SO_10_H]^+^ 514.0987; Found: 514.1003.

2,2′-((6-([4-Sulfamoylphenyl]amino)-1,3,5-triazine-2,4-diyl)bis(amino))diglutaric acid **11**

White solid; yield 67%. HILIC-DAD purity 99.22%. IR (KBr, υ_max_ cm^−1^): 3259 (br w, NH), 2967 (w), 2361 (w), 1713 (w, C=O), 1622 (m, C=C), 1589 (s), 1557 (s), 1495 (s), 1404 (m), 1318 (m, SO_2_NH_2_), 1240 (m, C-N), 1151 (s, SO_2_NH_2_), 1098 (w). ^1^H-NMR (400 MHz, DMSO-*d_6_*, 90 °C) δ: 9.12 (br s, 1H, Ar-**NH**), 7.88 (d, 2H, *J =* 8.9 Hz, 2 × Ar-H_a_), 7.69 (d, 2H, *J =* 8.8 Hz, 2 × Ar-H_b_), 6.39 (br s, 2H, SO_2_NH_2_), 5.65 (br s, 2H, 2 × **NH**-CH), 4.33 (t, 2H, *J =* 6.8 Hz, 2 × H-C(2)), 2.39–2.26 (m, 4H, *J =* 7.6 Hz, 2 × H-C(4)), 2.06-1.89 (m, 4H, 2 × H-C(3)). ^13^C-NMR (100.58 MHz, DMSO-*d_6_,* 90 °C) δ: 174.72, 174.23, 165.78, 164.58, 144.23, 136.69, 126.79, 119.14, 54.14, 32.25, 28.52. HRMS (ESI/QTOF, *m/z*): [M+H]^+^ Calcd. for [C_19_H_23_N_7_SO_10_H]^+^ 542.1300; Found: 542.1313.

### 3.3. Program and Software for the Computational Study of Sulfonamide Derivatives

All calculations were run on a personal computer packed with 3.2 GHz Intel i7-8700 CPU + 16 GB RAM and with Windows 10 as an operating system. ChemSketch [46] was used to draw 2D structures of the studied sulfonamide derivatives. The optimization of their 3D structures, based on the searching for a conformer with the lowest energy, was performed in Spartan ’14 program [47]. The calculation of all molecular descriptors was made using Dragon software [48]. The preparation of summarized data matrix as well as initial data pretreatment was performed by MS Excel 2007 [59]. Data preprocessing, which included the selection of the most relevant molecular descriptors by Data mining feature, altogether with the evaluation of the predictive models, was carried out using Statistica 12 package [49]. Docking to hCA II and IX was performed by combination of various Schrödinger software packages [55,56,57,58].

## 4. Conclusions

The newly proposed water based synthetic strategies represent an attractive solution for the preparation of 1,3,5-triazinyl-substituted benzenesulfonamide derivatives containing a symmetric pair of the amino acids with various polarities. When comparing the results obtained with the organic and water-based synthetic media, the later ones provide favorable conditions concerning the yield and purity of the products as well as the time of synthesis. The optimized green synthetic procedures were successfully applied for a series of new sulfonamide derivatives disubstituted with amino acids possessing polar uncharged (Asn, Gln, Ser, Thr) and hydrophobic (Ala, Trp, Tyr) side chain, so that a majority of the products could be isolated directly from the reaction mixtures with an acceptable purity (confirmed by the HPLC-ESI-DAD/QTOF/MS analytical products profiles). A substantial enhancement of purity and simplification of isolation procedure, essential especially for several more problematic derivatives (Tyr, Asn, Gln), was achieved using newly developed semi-preparative chromatography methods, applied for the crude synthetic products. High purities (>98%) and chemical structures of the desired chromatographically pretreated products 2–11 were confirmed by HPLC-DAD-MS, IR, and NMR spectra/profiles.

The QSAR analysis of new sulfonamide derivatives predicted impressive inhibition constants (K_I_s ranging in the interval of 8.4–29.6 nM) of Gln, Ser, Thr, and Ala derivatives against the tumor-associated membrane-bound hCA IX isoform, similar to or even lower than this one of acetazolamide. In the contrary, their inhibition activities against three physiological hCAs, namely cytosolic hCA I, II and transmembrane hCA IV were (very) low (235.8–10 000 nM). This provides (very) high inhibition selectivity of these derivatives towards the tumor-associated hCA IX isoform with a promising biomedical use. For example, the K_I_ ratio (hCA II/hCA IX) for Gln derivative was 139.1 and the ratios for some other physiological hCA isoforms were even higher (e.g., hCA I/hCA IX for Thr derivative was 555.1, hCA I/hCA IX for Gln derivative was 337.7, hCA IV/hCA IX for Ser derivative was 1149.4, hCA IV/hCA IX for Ala derivative was 1027.3). The structures of molecular complexes including hCA II or hCA IX with the Gln derivative, exhibiting the highest predicted selectivity ratio hCA II/hCA IX, were visualized using Maestro Software to reveal relevant binding interactions of the inhibitor in the active places of both enzymes.

The present work clearly demonstrated an importance of new green synthetic strategies for the preparation of new 1,3,5-triazinyl-aminobenzenesulfonamide conjugates disubstituted with amino acids of various polarity. Many of such compounds represent highly potent and selective hCA inhibitors, worthy of further investigation in oncological field.

## Figures and Tables

**Table 1:** Yield, purity, and characteristic impurities of the sulfonamide derivatives with amino acids of various polarities obtained from HPLC-ESI-DAD/QTOF/MS analysis of crude products.

(**a**)-the products with hydrophobic amino acids				
**Product (M_r_)**	**Parameter**	**Na_2_CO_3_**	**NaHCO_3_**	**TEA**
**2** Ala	yield of crude product (%)	99	73	48
(425.11 g/mol)	RP-HPLC-UV purity (%)	92.55	93.34	92.18
	MS [M + H]^+^ found	426.1196 (7.25 min)	426.1213 (7.29 min)	426.1210 (7.27 min)
	disubst. AA + OH (%)	x	x	1.19
**3** Phe	yield of crude product (%)	94	94	12
(577.17g/mol)	RP-HPLC-UV purity (%)	96.81	97.19	20.43
	MS [M + H]^+^ found	578.1829 (4.38 min)	578.1831 (4.39 min)	578.1862 (4.36 min)
**4** Tyr	yield of crude product (%)	87	77	1.7
(609.16 g/mol)	RP-HPLC-UV purity (%)	32.72	27.47	10.57
	MS [M + H]^+^ found	610.1727 (7.04 min)	610.1721 (7.04 min)	610.1766 (7.03 min)
	disubst. AA + OH (%)	32.23	1.06	x
	disubst. OH + OH (%)	2.7	x	1.22
	unreacted initial compounds (%)	x	23.34 (182.0812)	15.46 (182.0813)
**5** Trp	yield of crude product (%)	94	96	70
(655.19 g/mol)	RP-HPLC-UV purity (%)	93.12	87.44	77.82
	MS [M + H]^+^ found	656.2051 (5.5 min)	656.2046 (5.51 min)	656.2050 (5.51 min)
	disubst. AA + OH (%)	x	1.78	x
	disubst. OH + OH (%)	x	0.79	x
	unreacted initial compounds (%)	1.63 (205.0793)	8.94 (205.0975)	x
(**b**)-the products with polar uncharged amino acids				
**Product (M_r_)**	**Parameter**	**Na_2_CO_3_**	**NaHCO_3_**	**TEA**
**6** Asn	yield of crude product (%)	97	71	9.0
(511.12 g/mol)	RP-HPLC-UV purity (%)	49.66	88.47	16.31
	MS [M + H]^+^ found	512.1320 (9.63 min)	512.1320 (9.63 min)	512.1340 (9.65 min)
	disubst. AA + OH (%)	0.42	1.33	23.45
	monosubst. AA (%)	x	x	26.29
	disubst. OH + OH (%)	1.30	x	2.20
**7** Gln	yield of crude product (%)	78	68	18
(539.15 g/mol)	RP-HPLC-UV purity (%)	21.58	58.04	55.23
	MS [M + H]^+^ found	540.1635 (9 min)	540.1634 (9.01 min)	540.1650 (9.03 min)
	disubst. AA + OH (%)	9.42	3.41	2.64
	monosubst. AA (%)	4.91	19.76	3.42
	disubst. OH + OH (%)	1.17	x	x
**8** Ser	yield of crude product (%)	73	82	41
(457.42 g/mol)	RP-HPLC-UV purity (%)	87.09	94.86	4.08
	MS [M + H]^+^ found	458.1095 (9.13 min)	458.1095 (9.13 min)	458.1092 (9.13 min)
	disubst. AA + OH (%)	3.71	2.23	50.33
	monosubst. AA (%)	x	x	7.91
	disubst. OH + OH (%)	x	x	7.27
**9** Thr	yield of crude product (%)	93	92	14
(485.37 g/mol)	RP-HPLC-UV purity (%)	94.42	96.56	20.93
	MS [M + H]^+^ found	486.1411 (7.7 min)	486.1414 (7.71 min)	486.1415 (7.73 min)
	disubst. AA + OH (%)	1.50	1.43	22.17
	disubst. OH + OH (%)	x	x	4.25
(**c**)-the products with acidic (negatively charged) amino acids				
**Product (M_r_)**	**Parameter**	**Na_2_CO_3_**	**NaHCO_3_**	**TEA**
**10** Asp (513.09 g/mol)	yield of crude product (%)	90	46	16
	RP-HPLC-UV purity (%)	93.07	17.57	37.68
	MS [M + H]^+^ found	514.1003 (10.71 min)	514.1000 (10.74 min)	514.1023 (10.75 min)
	disubst. AA + OH (%)	5.37	21.96	6.92
	monosubst. AA (%)	x	50.99	x
	disubst. OH + OH (%)	0.32	1.36	9.99
**11** Glu (541.12 g/mol)	yield of crude product (%)	75	69	38
	RP-HPLC-UV purity (%)	89.53	16.68	58.00
	MS [M + H]^+^ found	542.1313 (10.76 min)	542.1310 (10.77 min)	542.1340 (10.77 min)
	disubst. AA + OH (%)	3.70	19.26	10.07
	monosubst. AA (%)	x	50.04	x
	disubst. OH + OH (%)	x	1.84	3.40

Main impurities, namely precursor **1** disubstituted with AA + OH, OH + OH, and monosubstituted with AA, as well as unreacted initial compounds were monitored for each product and their presence is indicated in these tables (1**a**–**c**).

**Table 2:** Summarized information about computed three-layer-perceptron architectures used for prediction of inhibitory data against selected human carboanhydrases (hCAs).

ANN		hCA I	hCA II	hCA IV	hCA IX
Architecture *	3-MLP	30-17-1	30-13-1	30-19-1	30-12-1
Activation function	Hidden layer	tanh	tanh	tanh	tanh
	Output layer	exp	log	log	log
Training set	Performance **	0.9643	0.9972	0.9994	0.9927
	Error ***	0.0039	0.0003	0.0001	0.0011
Validation set	Performance **	0.9411	0.9531	0.9441	0.9748
	Error ***	0.0111	0.0064	0.0097	0.0066

* Number of neurons: input layer-hidden layer-output layer; ** Performance-regression coefficient; *** Error—root mean squared error (RMSE).

**Table 3:** Comparison of measured and predicted inhibitory values for sulfonamide derivatives with a symmetric pair of amino acids.

Name	Linker	Model	hCA I (K_I_, nM)		hCA II (K_I_, nM)		hCA IV (K_I_, nM)		hCA IX (K_I_, nM)		hCA II/IX	
			Measured	Predicted	Measured	Predicted	Measured	Predicted	Measured	Predicted	Measured	Predicted
TSA Asp	0	Train	8259.8	8257.0	6219.1	6201.2	10000.0	10000.0	211.1	135.0	29.5	45.9
TSAM Asp	1	Train	3700.0	3682.6	5953.7	5693.6	10000.0	9999.6	2364.1	2159.9	2.5	2.6
TSAE Asp	2	Validation	10000.0	9866.0	5519.6	5931.6	10000.0	10000.0	25.8	44.7	213.9	132.7
TSA B-Ala	0	Train	673.0	364.2	368.0	235.8	9596.0	9990.0	8.9	12.5	41.3	18.9
TSAM B-Ala	1	Train	655.4	309.4	661.6	239.6	4476.0	4519.5	1818.5	1395.9	0.4	0.2
TSAE B-Ala	2	Train	960.7	1396.7	892.1	752.2	10000.0	9999.8	134.2	8.5	6.6	88.7
TSA Glu	0	Validation	9260.8	8976.3	7125.0	6203.0	10000.0	9999.8	202.4	65.7	35.2	94.4
TSAM Glu	1	Validation	6214.1	7938.1	695.2	771.3	4125.8	5622.2	193.2	19.5	3.6	39.6
TSAE Glu	2	Train	10000.0	10035.4	2284.3	2269.0	10000.0	9910.8	27.1	28.3	84.3	80.3
TSA Gly	0	Train	4550.0	277.0	376.0	235.7	10000.0	9978.9	8.4	8.8	44.8	26.8
TSAM Gly	1	Validation	9362.4	5603.6	478.4	235.7	867.3	1651.2	145.8	10.5	3.3	22.4
TSAE Gly	2	Train	4023.8	4032.9	428.1	235.7	3415.2	3465.8	330.4	8.4	1.3	28.1
TSA Ile	0	Train	10000.0	9724.8	2948.9	2952.7	4021.2	4024.9	92.6	47.1	31.8	62.7
TSAM Ile	1	Train	664.5	914.8	628.5	807.6	76.3	154.9	164.3	22.1	3.8	36.5
TSAE Ile	2	Train	7337.2	7292.8	1556.2	1644.5	1421.3	1301.5	189.3	19.9	8.2	82.8
TSA Leu	0	Train	4191.9	4392.1	4528.8	4505.6	2380.9	2482.7	24.0	25.2	188.7	178.6
TSAM Leu	1	Validation	96.9	387.6	396.0	588.8	516.8	537.2	167.3	288.6	2.4	2.0
TSAE Leu	2	Train	4854.5	4960.0	912.5	511.3	367.1	171.1	123.8	32.0	7.4	16.0
TSA Met	0	Train	346.7	798.1	803.7	565.5	3294.0	3040.8	2222.2	1960.4	0.4	0.3
TSAM Met	1	Train	531.3	459.8	3170.6	3153.4	45.6	64.0	1274.9	1962.3	2.5	1.6
TSAE Met	2	Validation	4527.8	1988.3	5017.9	4957.6	2336.1	60.1	2592.4	1951.0	1.9	2.5
TSA Phe	0	Train	305.4	210.1	866.1	870.8	9387.0	9998.7	191.5	131.5	4.5	6.6
TSAM Phe	1	Train	67.1	364.1	235.7	394.3	61.9	47.9	119.6	107.9	2.0	3.7
TSAE Phe	2	Train	4893.8	4847.3	6161.8	5956.5	374.5	47.5	223.1	22.9	27.6	259.8
TSA Pro	0	Validation	87.0	188.4	3112.8	3168.2	9315.0	8607.2	295.0	56.1	10.6	56.4
TSAM Pro	1	Train	256.4	204.1	773.1	759.2	350.4	329.7	265.5	158.4	2.9	4.8
TSAE Pro	2	Validation	958.4	398.8	1070.8	667.0	2206.9	719.9	25.7	47.9	41.7	13.9
TSA Val	0	Train	398.7	498.5	5335.4	5503.5	10000.0	9942.7	2111.1	2241.1	2.5	2.5
TSAM Val	1	Validation	377.0	308.8	839.3	1050.1	656.8	418.9	1371.8	457.2	0.6	2.3
TSAE Val	2	Train	932.2	613.9	804.1	857.2	476.3	677.7	130.9	179.2	6.1	4.8

Validation was used for experimental determination of inhibition data and calculations of predicted values were made by using artificial neural networks. TSA = triazinyl-aminobenzenesulfonamide; TSAM = triazinyl-aminomethylbenzenesulfonamide; TSAE = triazinyl-aminoethylbenzenesulfonamide; 0 = amino; 1 = aminomethyl; 2 = aminoethyl.

**Table 4:** Predicted inhibitory values for new sulfonamide derivatives disubstituted with proteinogenic amino acids possessing polar neutral and hydrophobic side chains.

	K_I_ (nM)				Selectivity
TSA Derivative	hCA I	hCA II	hCA IV	hCA IX	hCA II/IX
**2** Ala	239.6	235.8	8629.1	8.4	28.0
**4** Tyr	376.0	6198.0	9998.5	192.9	32.1
**5** Trp	10085.9	6217.8	9837.2	2218.0	2.8
**6** Asn	8892.8	924.6	9930.3	720.1	1.3
**7** Gln	9996.2	4111.2	2328.9	29.6	139.1
**8** Ser	4711.8	250.8	10000.0	8.7	28.8
**9** Thr	7271.6	555.3	3099.5	13.1	42.3

TSA = triazinyl-aminobenzenesulfonamide; K_I_ = inhibition constant; All derivatives contain a symmetric pair of given amino acid.

**Table 5:** Chromatographic conditions used for purification of the crude products by preparative LC.

Product	Stationary Phase	Mobile Phase A	Mobile Phase B	%B
**2** Ala	RP-C18	100 mM NH_4_HCO_3_	Methanol	12.5
**3** Phe	RP-C18	100 mM NH_4_HCO_3_	Methanol	45.0
**4** Tyr	RP-C18	100 mM NH_4_HCO_3_	Methanol	25.0
**5** Trp	RP-C18	100 mM NH_4_HCO_3_	Methanol	40.0
**6** Asn	RP-C18	50 mM NH_4_HCO_3_	Methanol	5.0
**7** Gln	RP-C18	100 mM NH_4_HCO_3_	Methanol	7.5
**8** Ser	RP-C18	50 mM NH_4_HCO_3_	Methanol	5.0
**9** Thr	RP-C18	100 mM NH_4_HCO_3_	Methanol	10.0
**10** Asp	HILIC	100 mM NH_4_HCO_3_	Acetonitrile	72.5
**11** Glu	HILIC	100 mM NH_4_HCO_3_	Acetonitrile	75.0

## Supplementary Materials

Supplementary Materials can be found at https://www.mdpi.com/1422-0067/21/10/3661/s1.

## Abbreviations

hCA	human carbonic anhydrase
hCAi	human carbonic anhydrase inhibitors
PG	proteoglycan
HIF	hypoxia inducible factor
VHL	Von Hippel-Lindau (tumor suppressor)
QSAR	Quantitative structure-activity relationship
MMFF	Molecular mechanics force field
ANN	Artificial neural networks
3-MLP	3-layers of Multilayer perceptron neural networks
RMSE	root mean squared error
TSA	triazinyl-aminobenzenesulfonamide derivative
TSAM	triazinyl-aminomethylbenzenesulfonamide derivative
TSAE	triazinyl-aminoethylbenzenesulfonamide derivative
