# Supplementary material for: Synthetic Strategies and Computational Inhibition Activity Study for Triazinyl-Substituted Benzenesulfonamide Conjugates with Polar and Hydrophobic Amino Acids as Inhibitors of Carbonic Anhydrases
Source: Int J Mol Sci. 2020 May 22;21(10):3661. doi: 10.3390/ijms21103661 (PMC7279466; doi:10.3390/ijms21103661)
Supplement: Supplementary file 1 [file ijms-21-03661-s001.pdf]

International Journal of *Molecular Sciences*

MDPI

Supplementary Material for:

Synthetic Strategies and Computational Inhibition Activity Study for Triazinyl-substituted Benzenesulfonamide Conjugates with Polar and Hydrophobic Amino Acids as Inhibitors of Carbonic Anhydrases

Mária Bodnár Mikulová[1], Dáša Kružlicová[1], Daniel Pecher[1,2], Claudiu T. Supuran[3], Peter Mikuš[1,2,*]

[1] Department of Pharmaceutical Analysis and Nuclear Pharmacy, Faculty of Pharmacy, Comenius University in Bratislava, Odbojárov 10, SK-832 32 Bratislava, Slovakia; mikulova43@uniba.sk (M.M.); pecher1@uniba.sk (D.P.); kruzlicova@fpharm.uniba.sk (D.K.)

[2] Toxicological and Antidoping Center, Faculty of Pharmacy, Comenius University in Bratislava, Odbojárov 10, SK-832 32 Bratislava, Slovakia

[3] Neurofarba Department, Section of Pharmaceutical and Nutriceutical Sciences, University of Florence, Italy; claudiu.supuran@unifi.it (C.S.).

* Correspondence: mikus@fpharm.uniba.sk (P.M.); ORCID 0000-0001-5755-9970

S1. 2,2′-((6-([4-Sulfamoylphenyl]amino)-1,3,5-triazine-2,4-diyl)bis(amino))dipropanoic acid **2** and its IR (a), ^{1}H (b), ^{13}C (c) NMR and DAD/MS (d) spectra

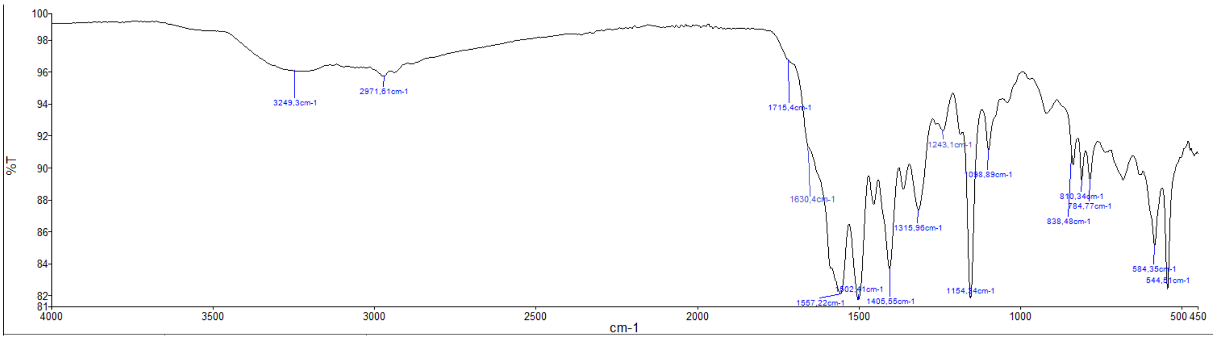

Figure S1a. IR spectra of product **2.**

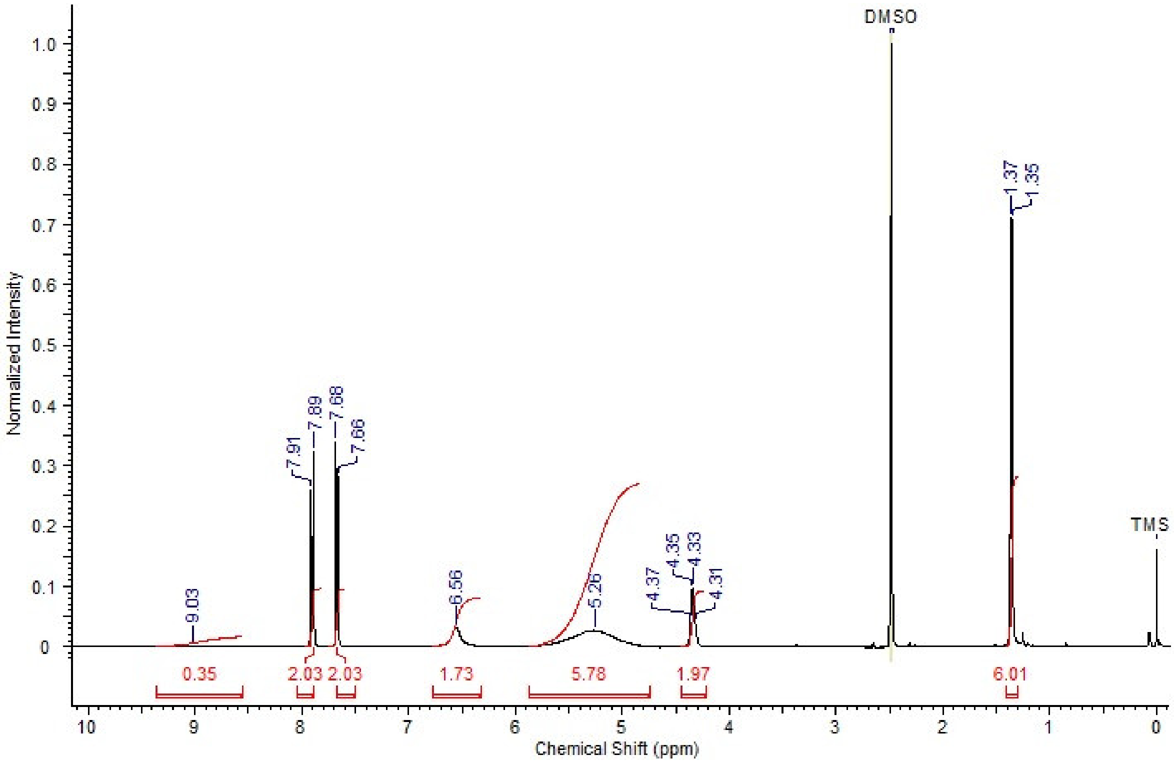

Figure S1b. ^{1}H NMR spectra of product **2.**

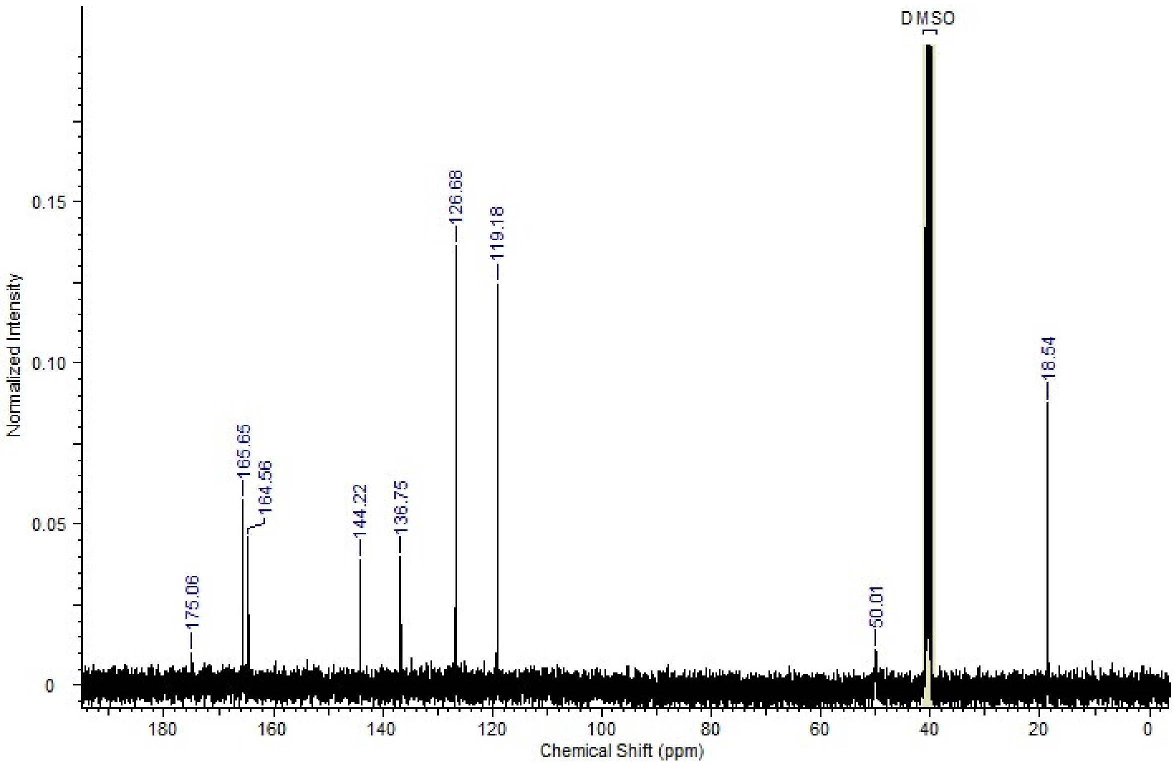

Figure S1c. ^{13}C NMR spectra of product **2.**

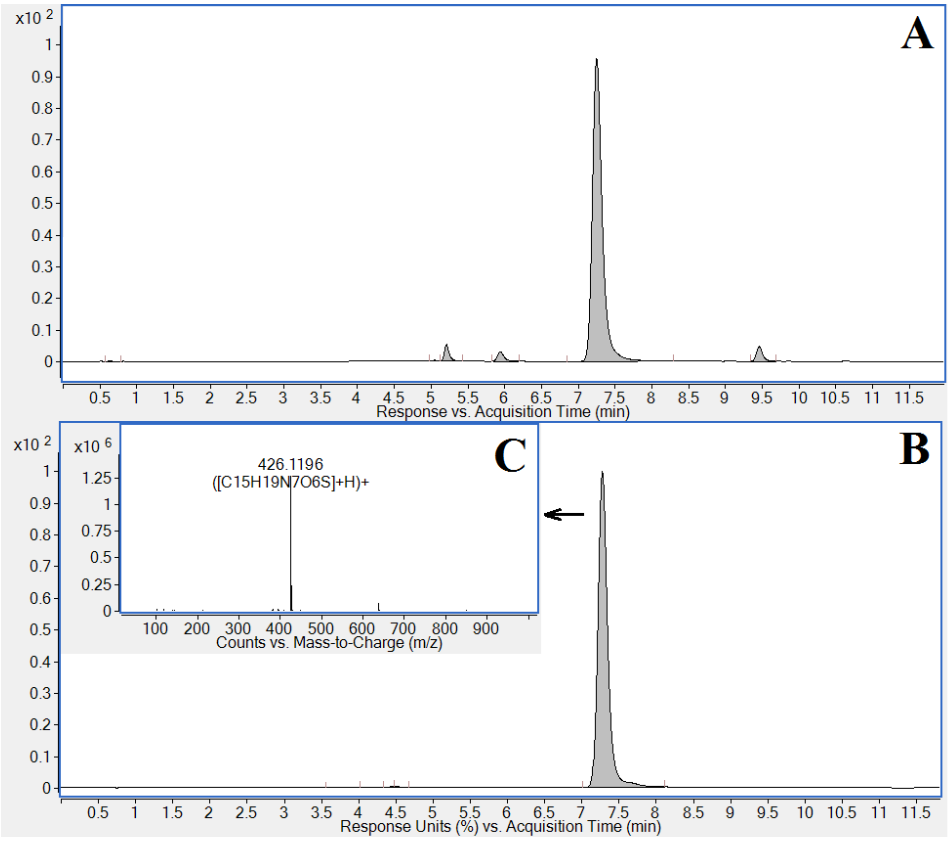

Figure S1d. HILIC-DAD-QTOF analysis of product **2**. Purity profile (HILIC-DAD - 254 nm) before (panel A) and after (panel B) semi-preparative chromatography. Panel C – MS spectrum of the desired compound (the major peak from UV chromatogram).

S2. 2,2′-((6-([4-Sulfamoylphenyl]amino)-1,3,5-triazine-2,4-diyl)bis(amino))di(3-phenylpropanoic acid) **3** and its IR (a), ^{1}H (b), ^{13}C (c) NMR and DAD/MS (d) spectra

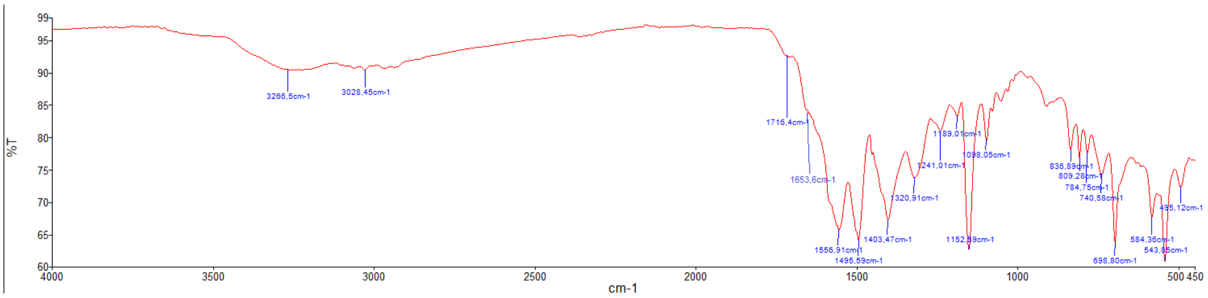

Figure S2a. IR spectra of product **3.**

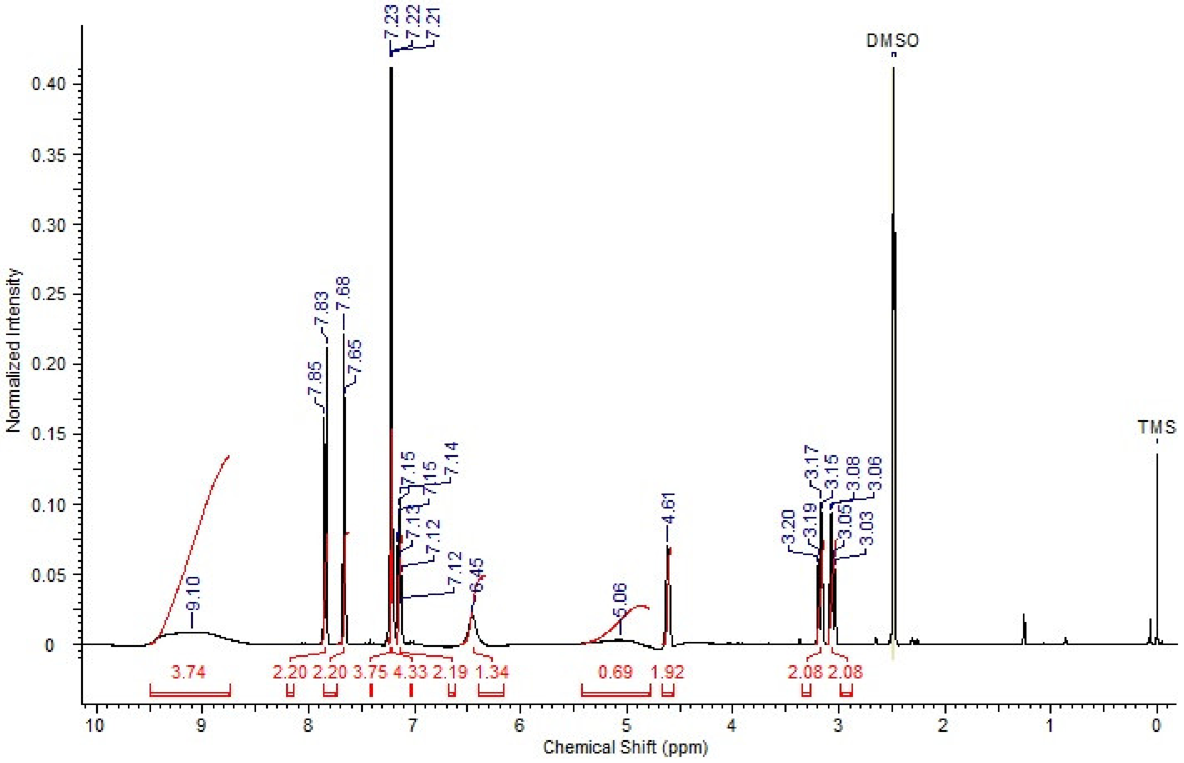

Figure S2b. ^{1}H NMR spectra of product **3.**

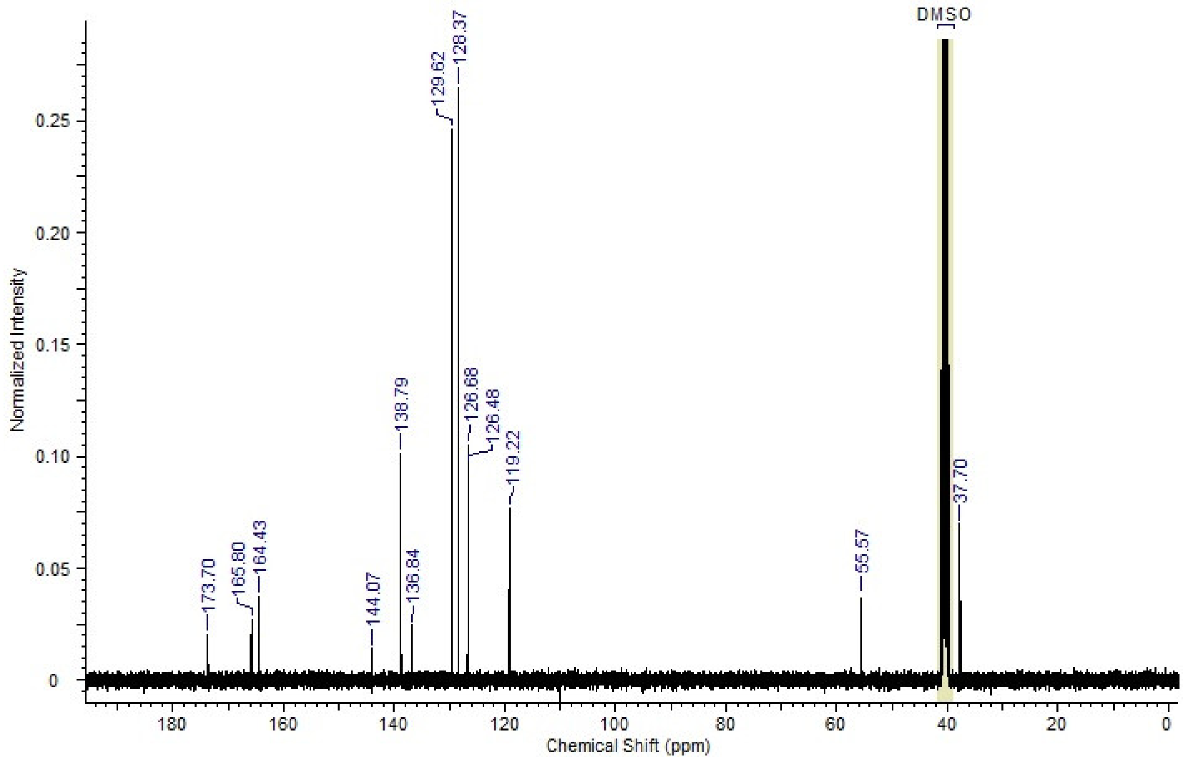

Figure S2c. ^{13}C NMR spectra of product **3.**

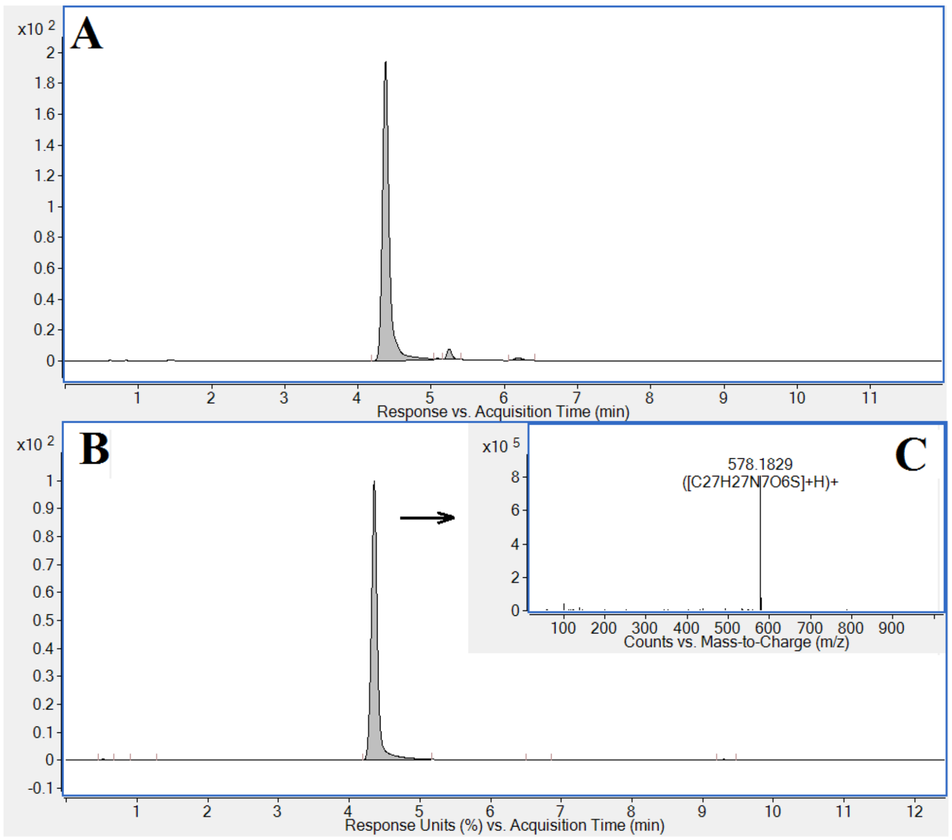

Figure S2d. HILIC-DAD-QTOF analysis of product **3**. Purity profile (HILIC-DAD - 254 nm) before (panel A) and after (panel B) semi-preparative chromatography. Panel C – MS spectrum of the desired compound (the major peak from UV chromatogram).

S3.

2,2′-((6-([4-Sulfamoylphenyl]amino)-1,3,5-triazine-2,4-diyl)bis(amino))di(3-(4-hydroxyphenyl)propanoic acid) **4** and its IR (a), ^{1}H (b), ^{13}C (c) NMR and DAD/MS (d) spectra

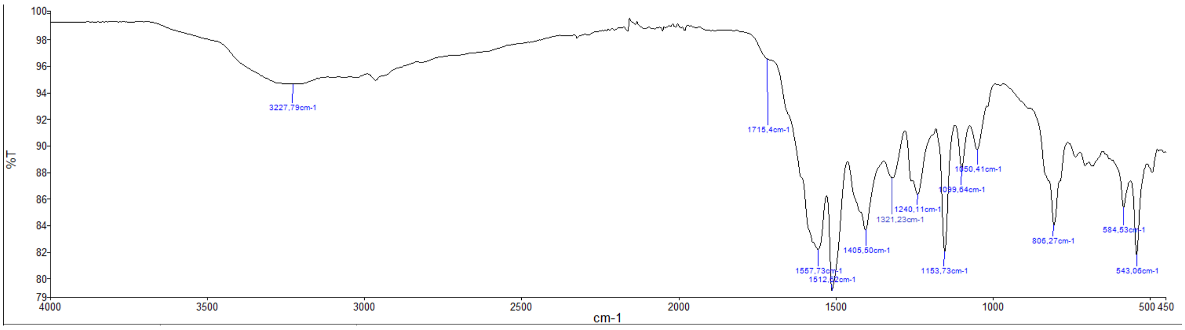

Figure S3a. IR spectra of product **4.**

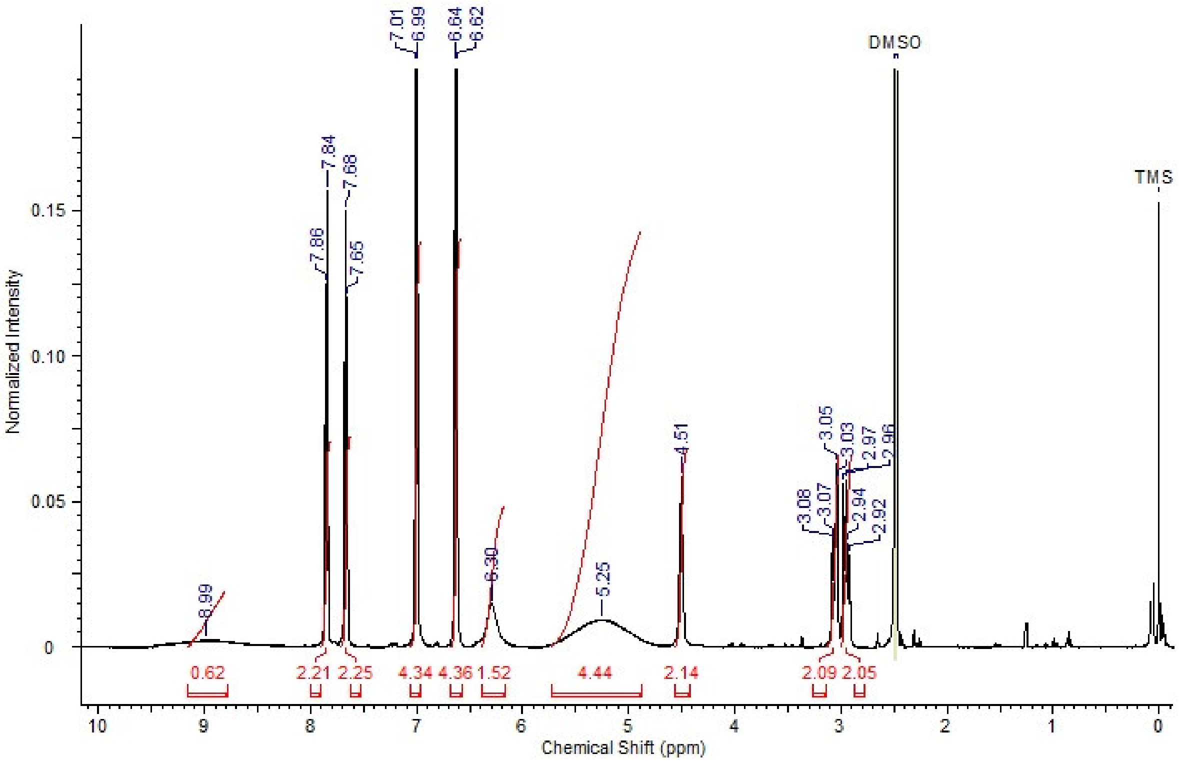

Figure S3b. ^{1}H NMR spectra of product **4.**

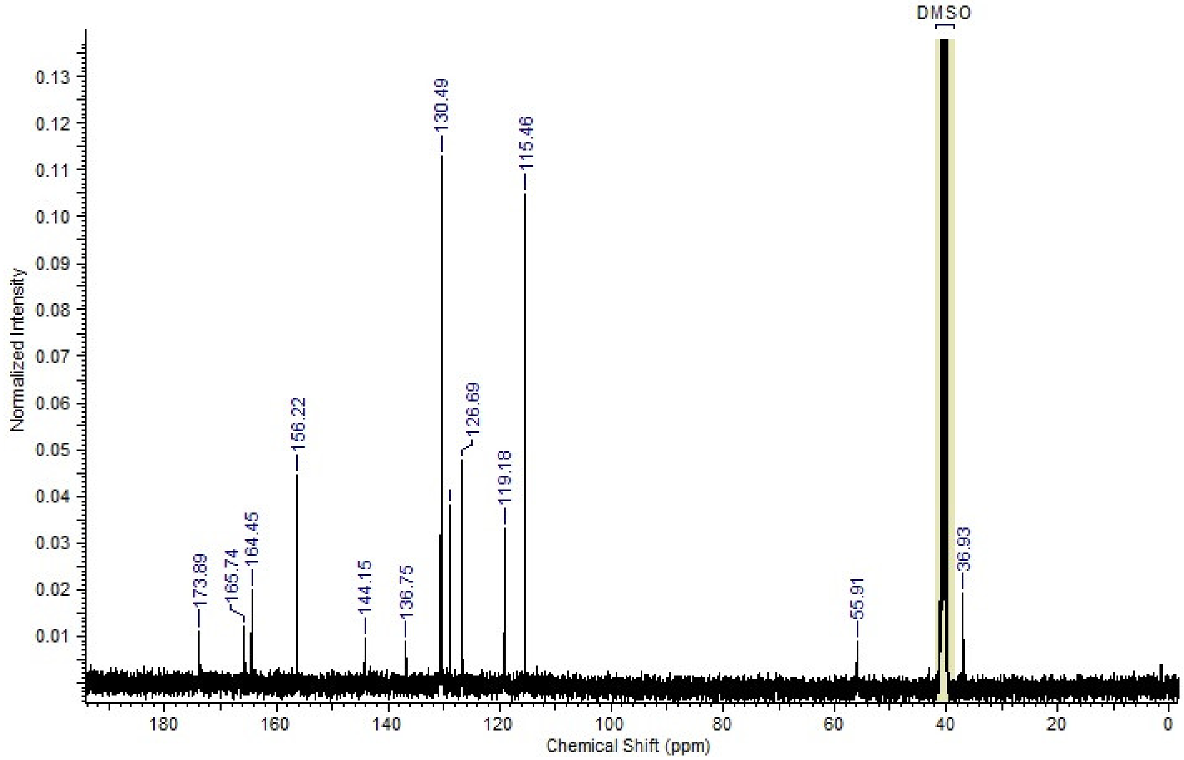

Figure S3c. ^{13}C NMR spectra of product **4.**

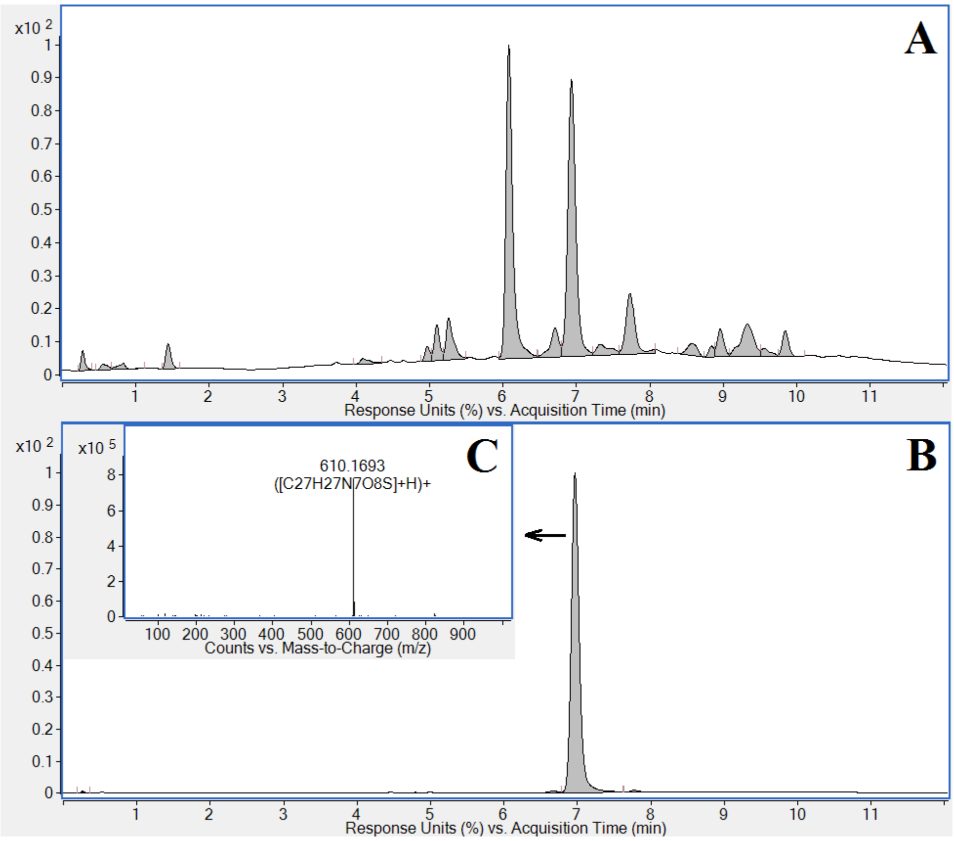

Figure S3d. HILIC-DAD-QTOF analysis of product **4**. Purity profile (HILIC-DAD - 254 nm) before (panel A) and after (panel B) semi-preparative chromatography. Panel C – MS spectrum of the desired compound (the major peak from UV chromatogram).

S4.
2,2′-((6-([4-Sulfamoylphenyl]amino)-1,3,5-triazine-2,4-diyl)bis(amino))di-(3-(1*H*-indol-3-yl)propanoic acid) **5** and its IR (a), ^{1}H (b), ^{13}C (c) NMR and DAD/MS (d) spectra

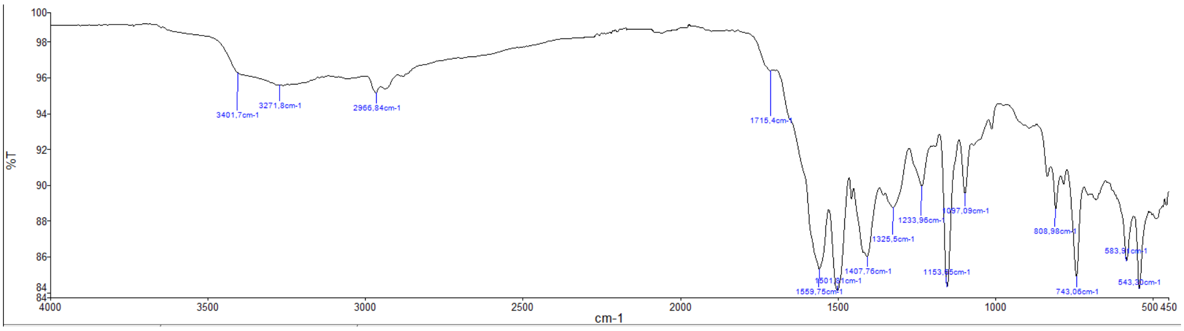

Figure S4a. IR spectra of product **5.**

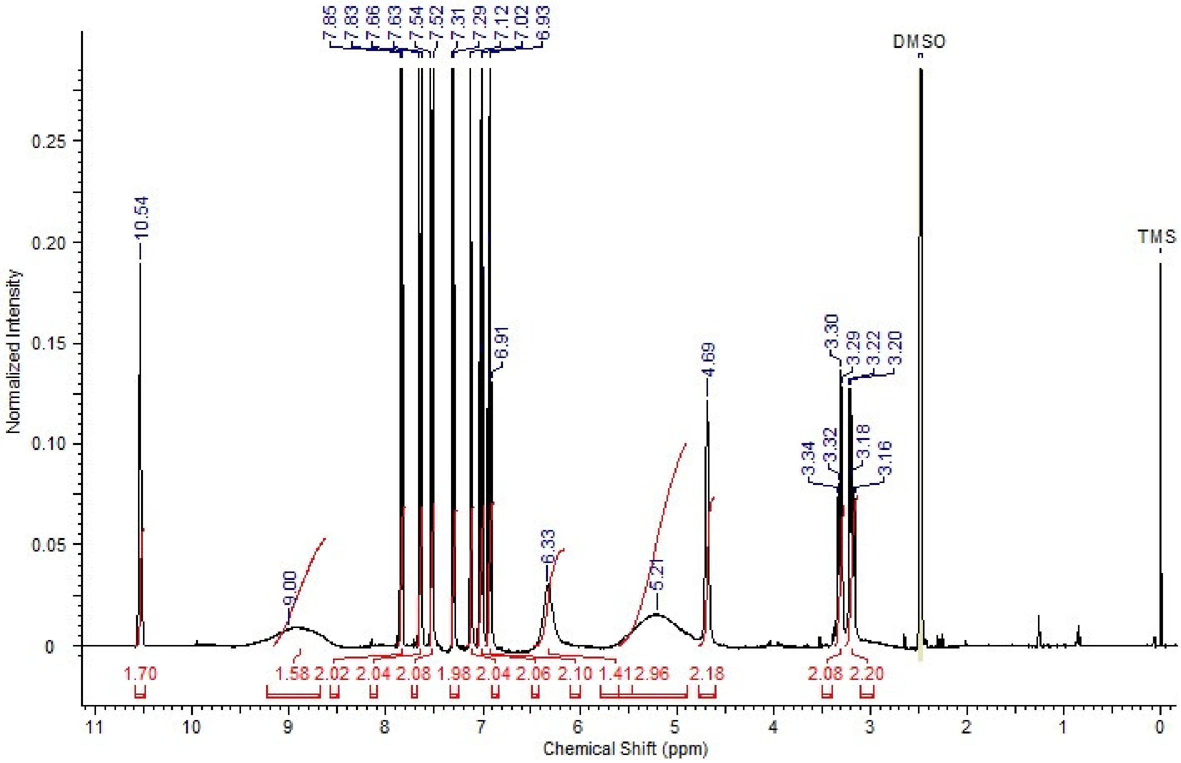

Figure S4b. ^{1}H NMR spectra of product **5.**

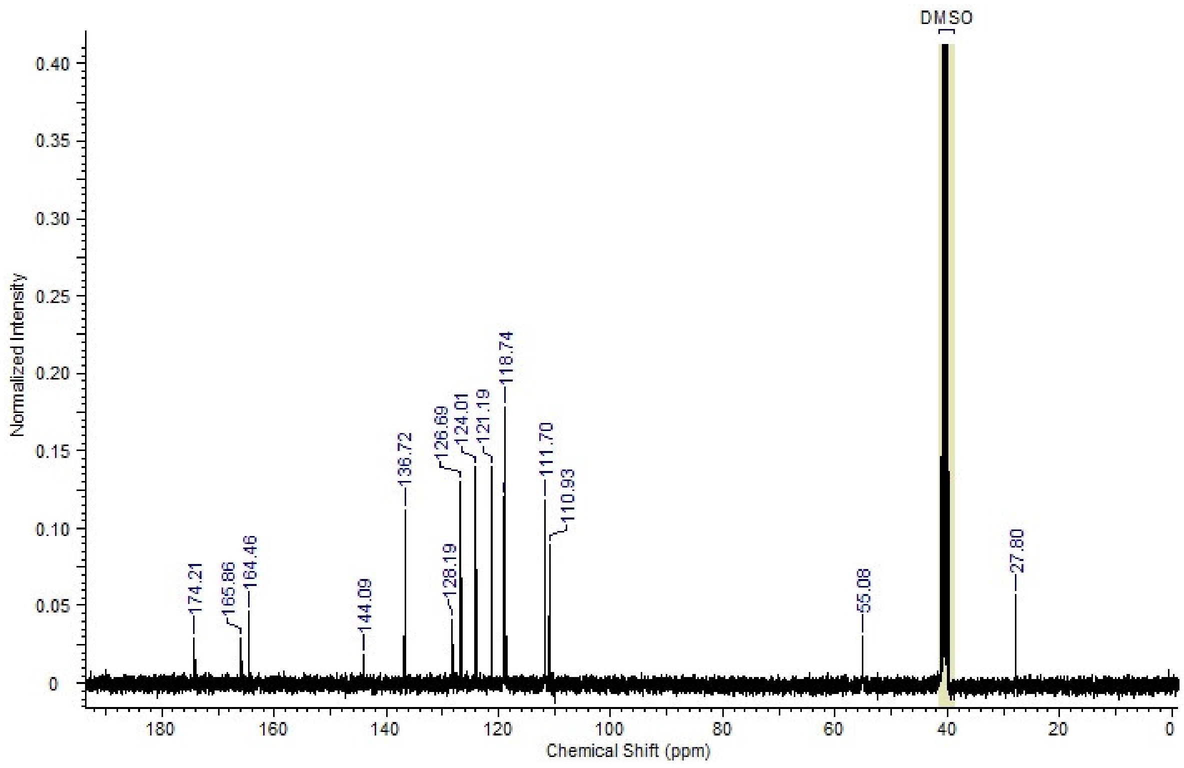

Figure S4c. ^{13}C NMR spectra of product **5.**

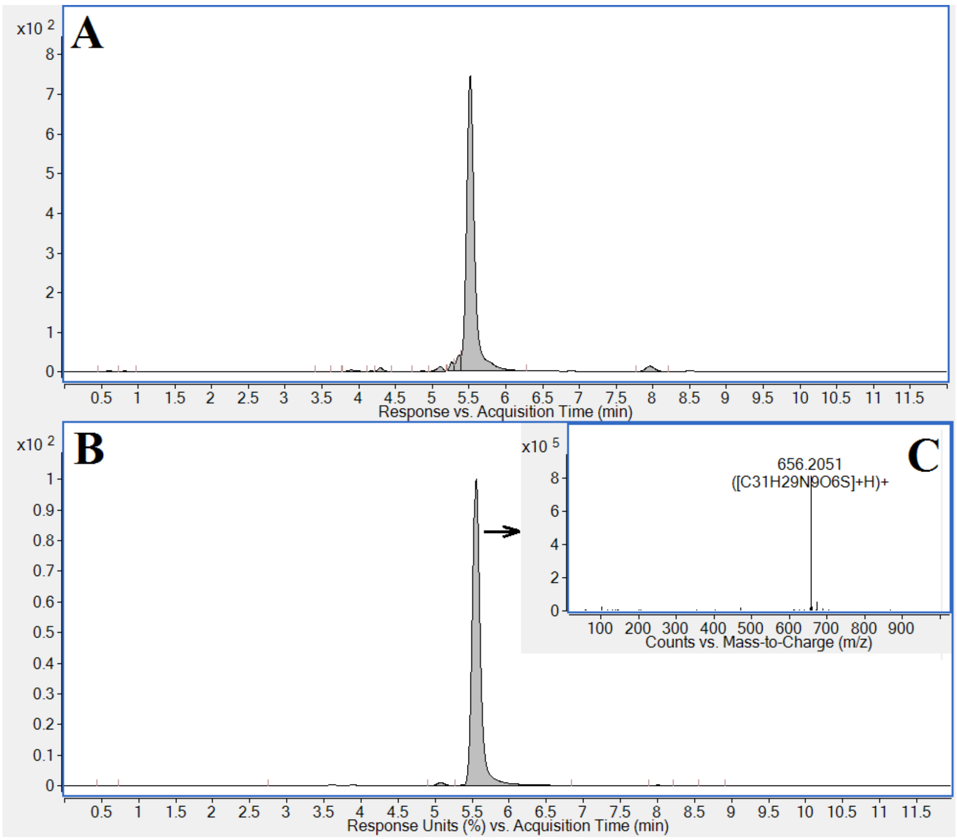

Figure S4d. HILIC-DAD-QTOF analysis of product **5**. Purity profile (HILIC-DAD - 254 nm) before (panel A) and after (panel B) semi-preparative chromatography. Panel C – MS spectrum of the desired compound (the major peak from UV chromatogram).

S5. 2,2′-((6-([4-Sulfamoylphenyl]amino)-1,3,5-triazine-2,4-diyl)bis(amino))di(4-amino-4-oxobutanoic acid) **6** and its IR (a), ^{1}H (b), ^{13}C (c) NMR and DAD/MS (d) spectra.

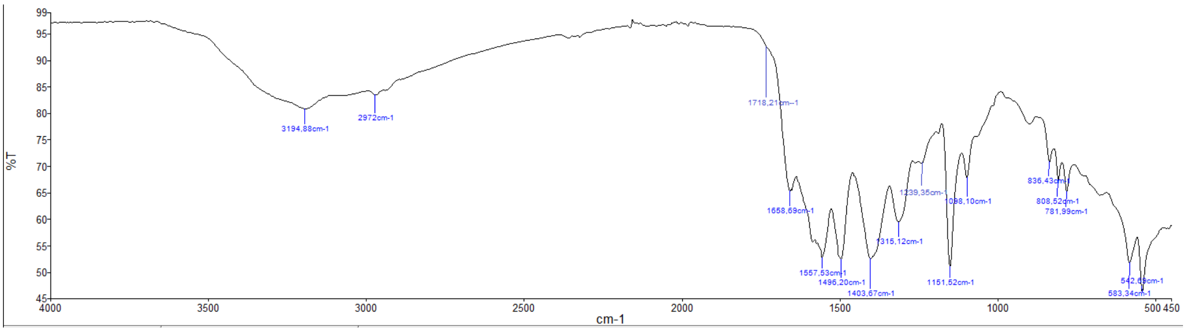

Figure S5a. IR spectra of product **6.**

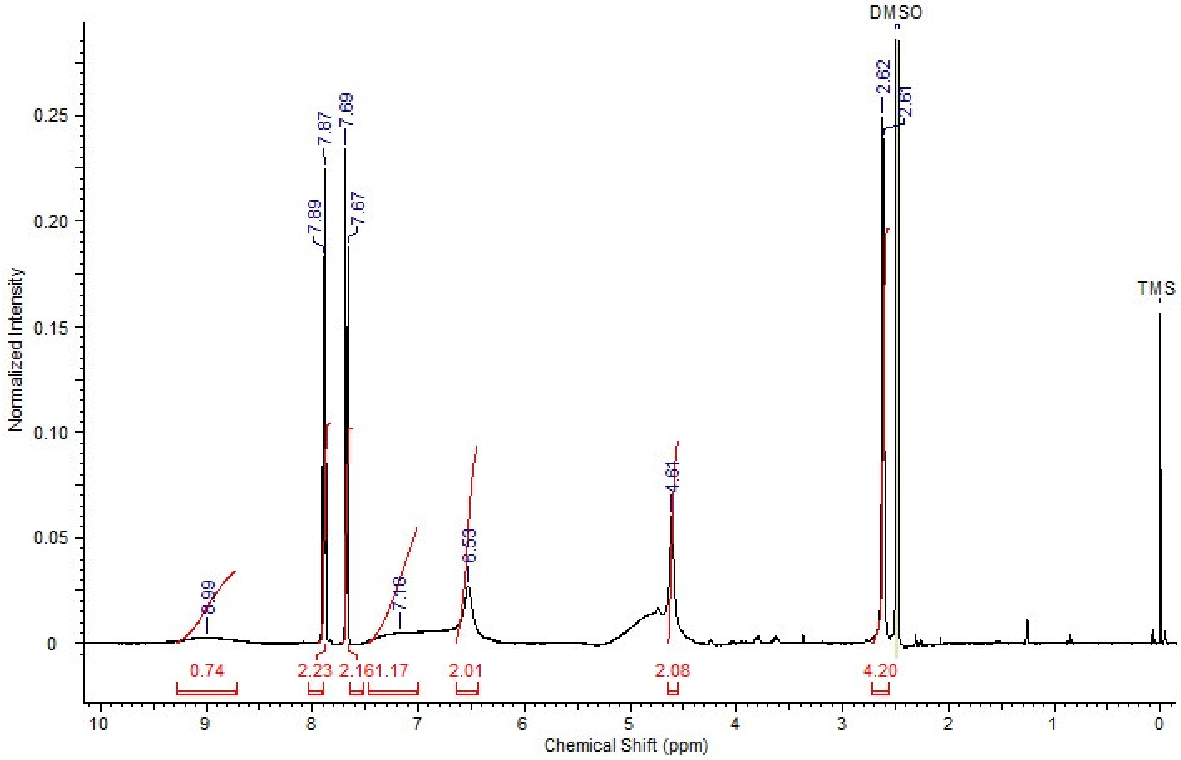

Figure S5b. ^{1}H NMR spectra of product **6.**

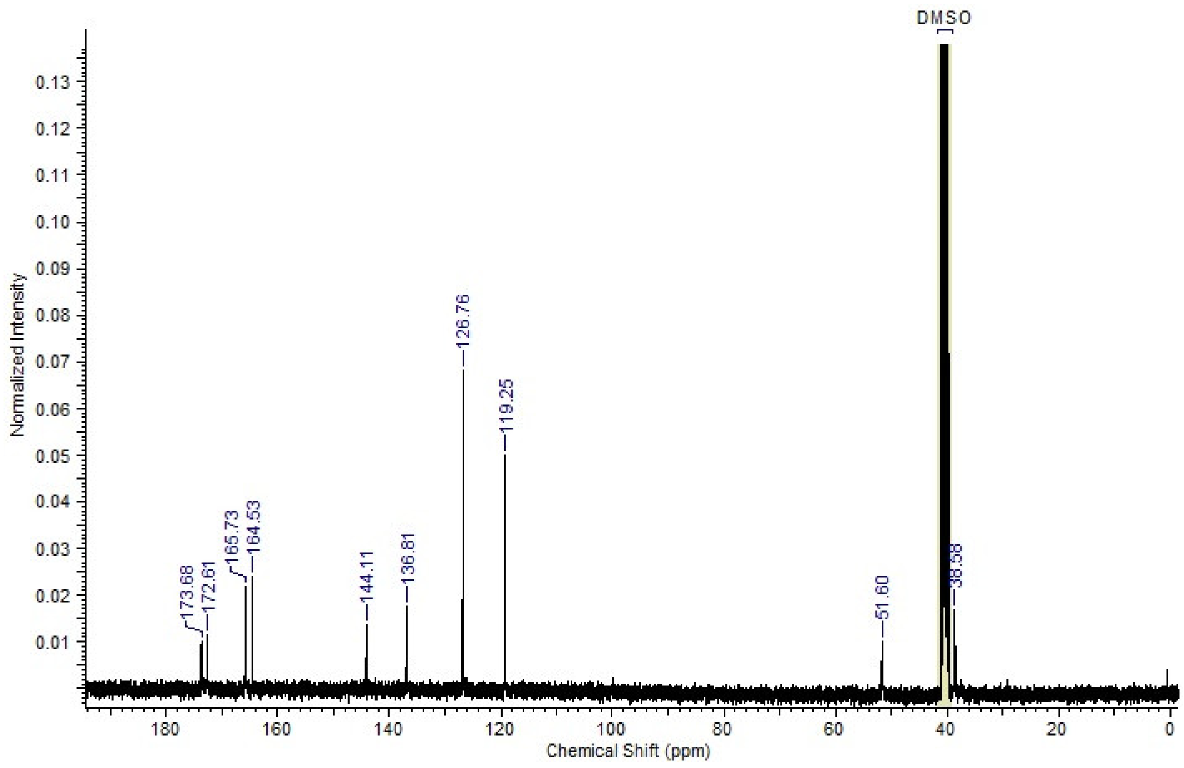

Figure S5c. ^{13}C NMR spectra of product **6.**

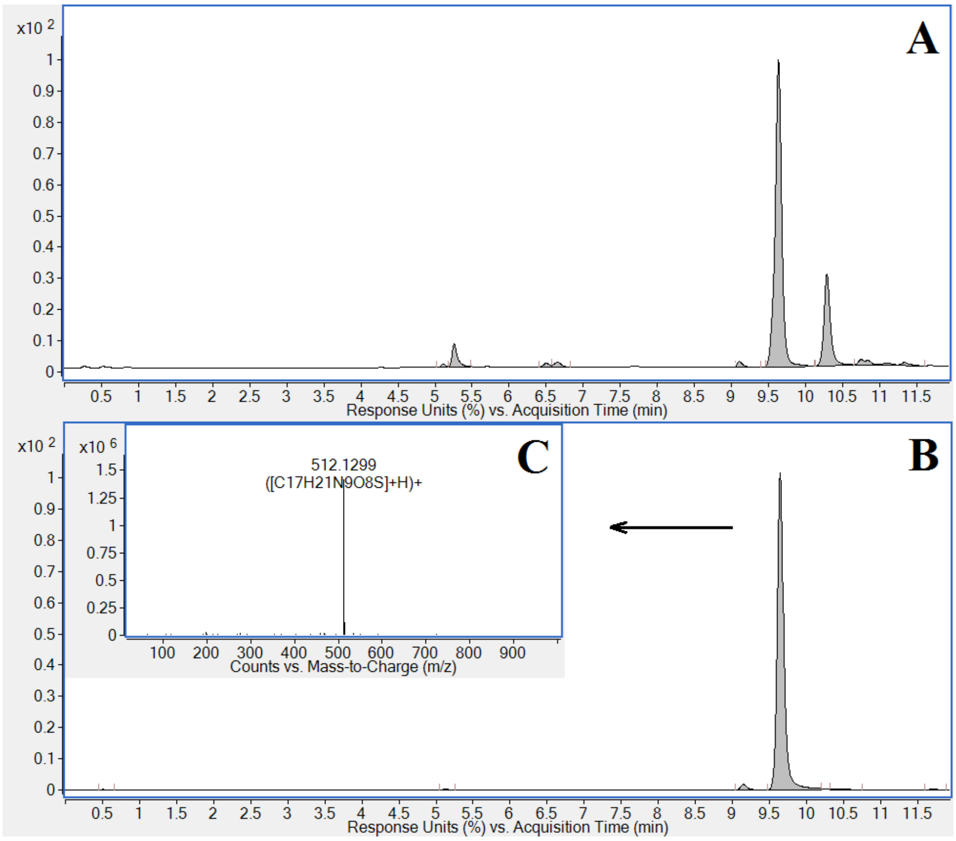

Figure S5d. HILIC-DAD-QTOF analysis of product **6**. Purity profile (HILIC-DAD - 254 nm) before (panel A) and after (panel B) semi-preparative chromatography. Panel C – MS spectrum of the desired compound (the major peak from UV chromatogram).

S6.

2,2′-((6-([4-Sulfamoylphenyl]amino)-1,3,5-triazine-2,4-diyl)bis(amino))di(5-amino-5-oxopentanoic acid) **7** and its IR (a), ^{1}H (b), ^{13}C (c) NMR and DAD/MS (d) spectra.

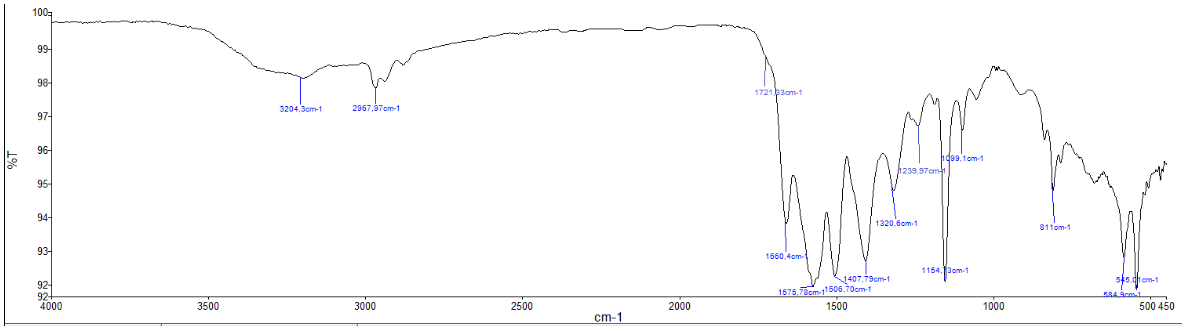

Figure S6a. IR spectra of product **7.**

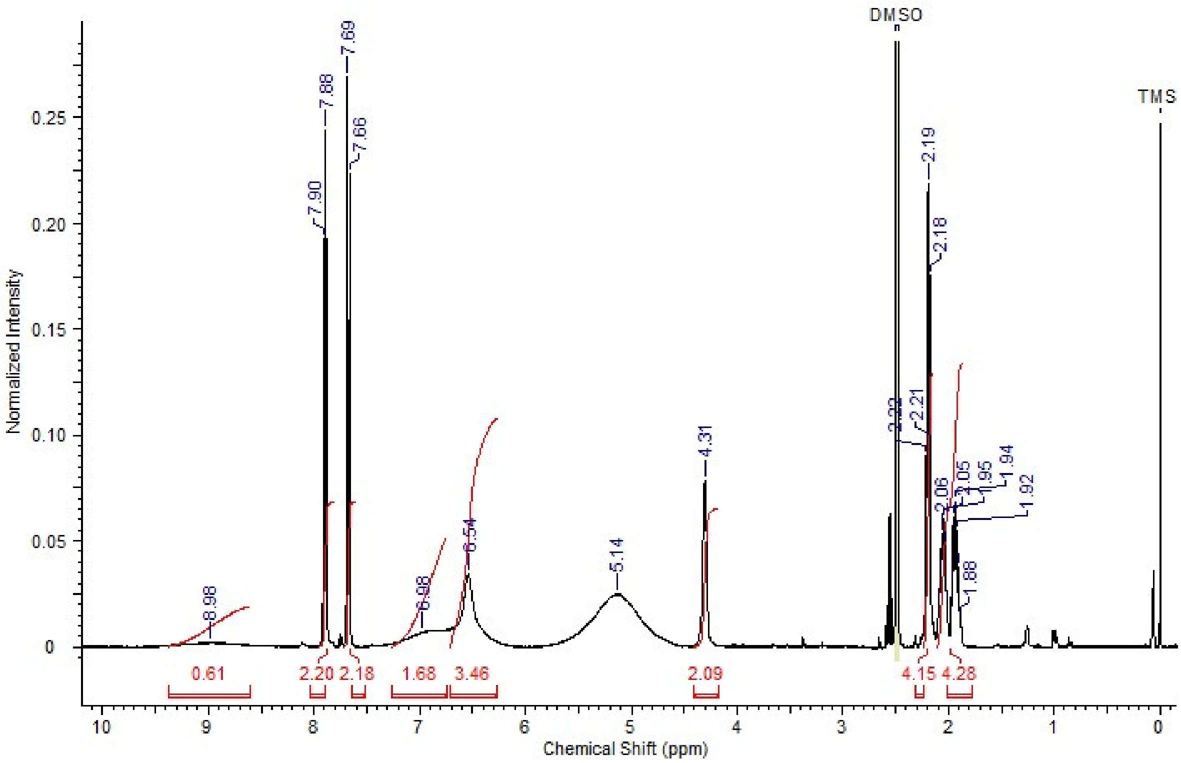

Figure S6b. ^{1}H NMR spectra of product **7.**

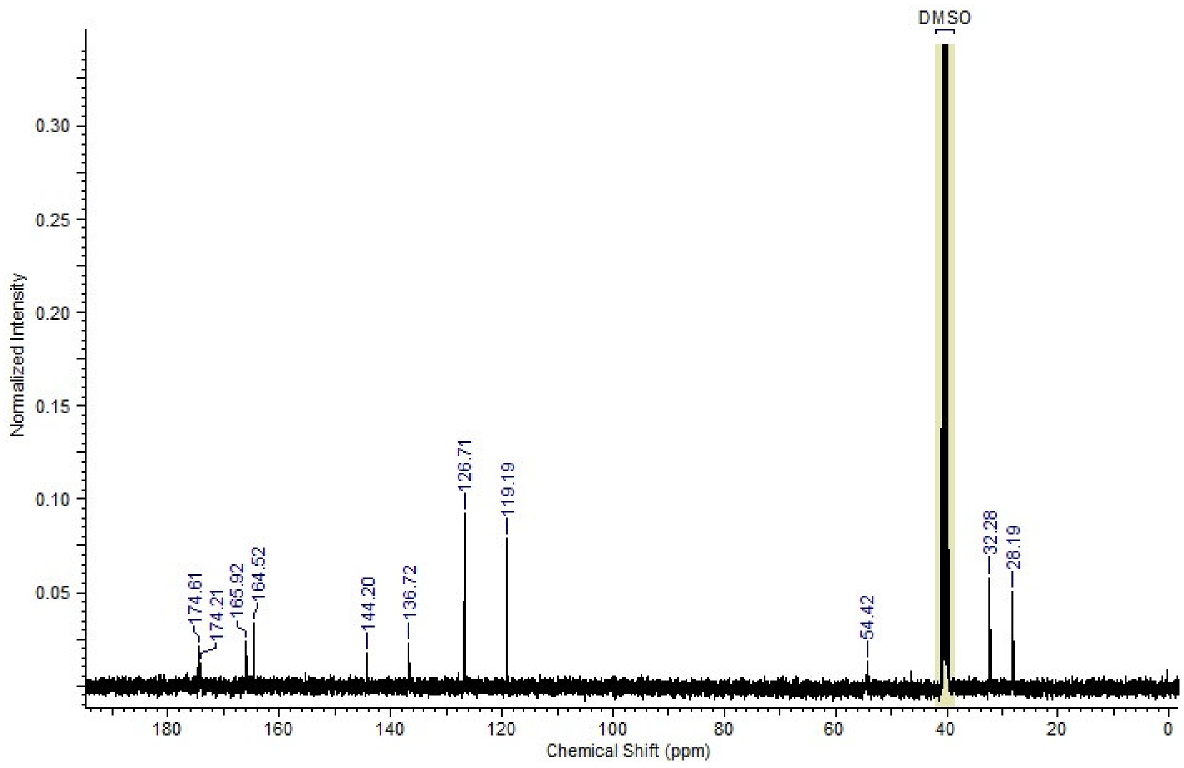

Figure S6c. ^{13}C NMR spectra of product **7.**

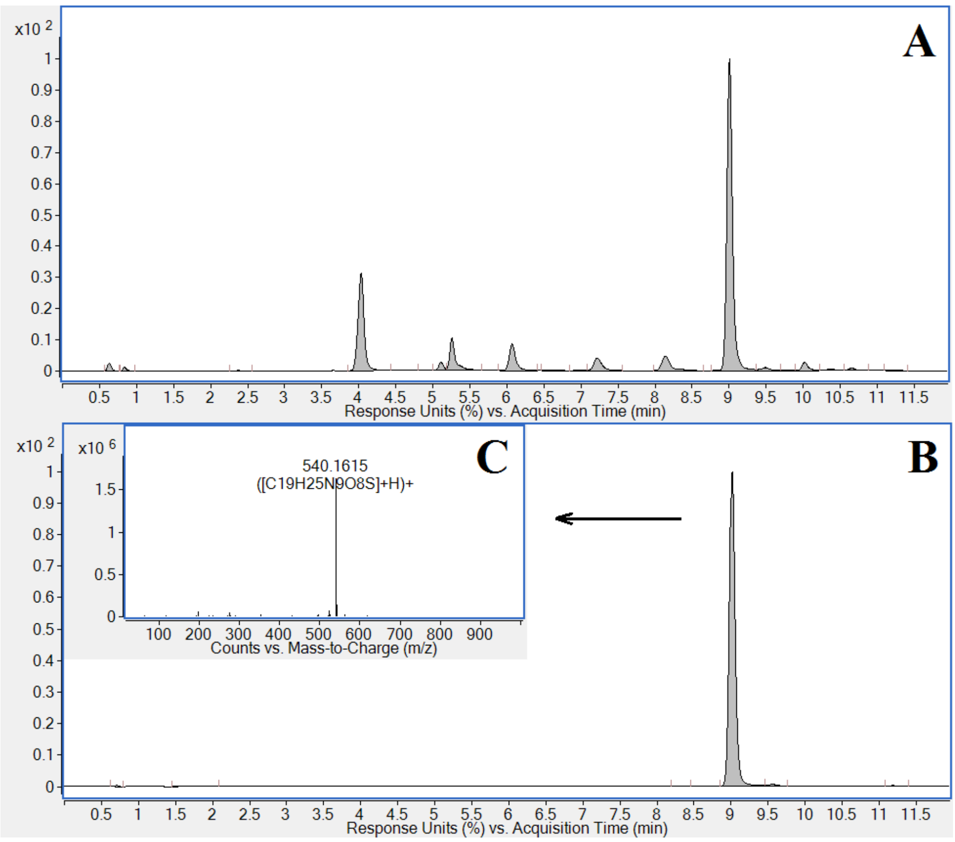

Figure S6d. HILIC-DAD-QTOF analysis of product **7**. Purity profile (HILIC-DAD - 254 nm) before (panel A) and after (panel B) semi-preparative chromatography. Panel C – MS spectrum of the desired compound (the major peak from UV chromatogram).

S7. 2,2′-((6-([4-Sulfamoylphenyl]amino)-1,3,5-triazine-2,4-diyl)bis(amino))di(3-hydroxypropanoic acid) **8** and its IR (a), ^{1}H (b), ^{13}C (c) NMR and DAD/MS (d) spectra

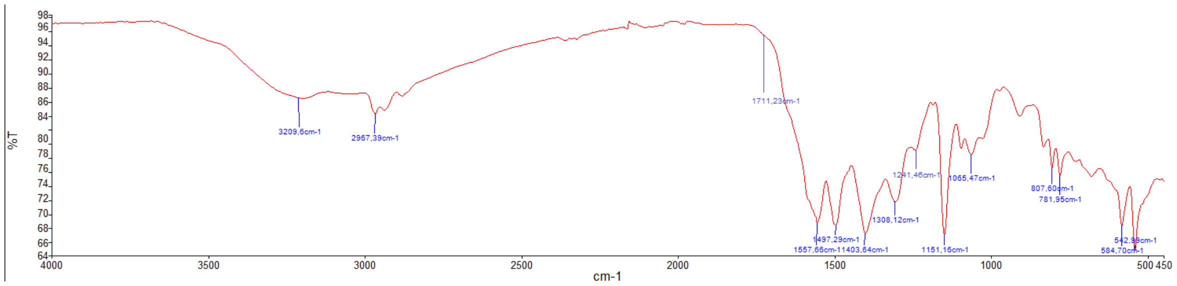

Figure S7a. IR spectra of product **8.**

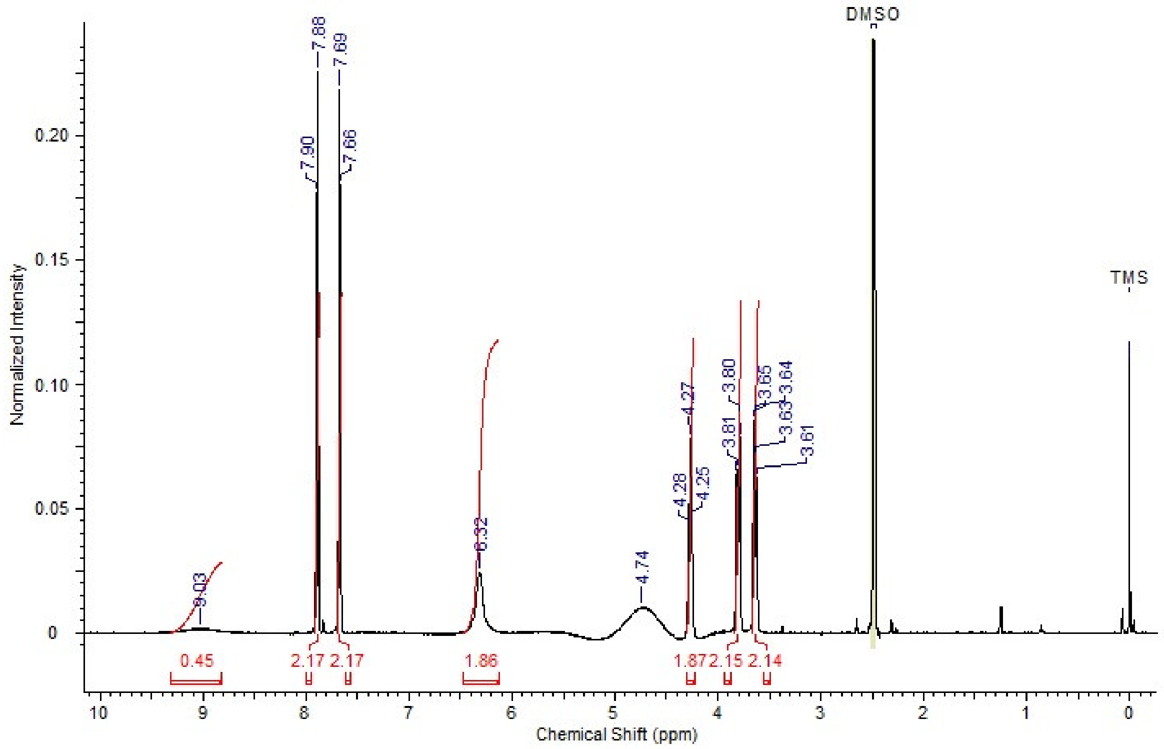

Figure S7b. ^{1}H NMR spectra of product **8.**

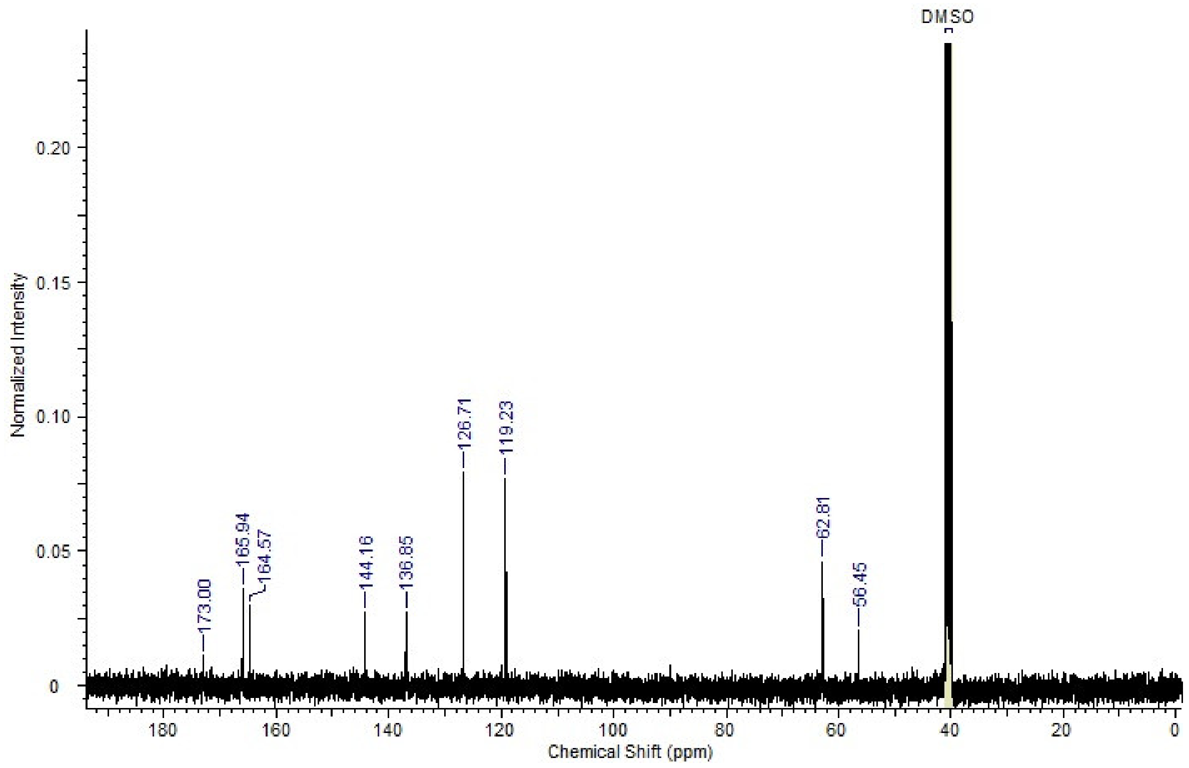

Figure S7c. ^{13}C NMR spectra of product **8.**

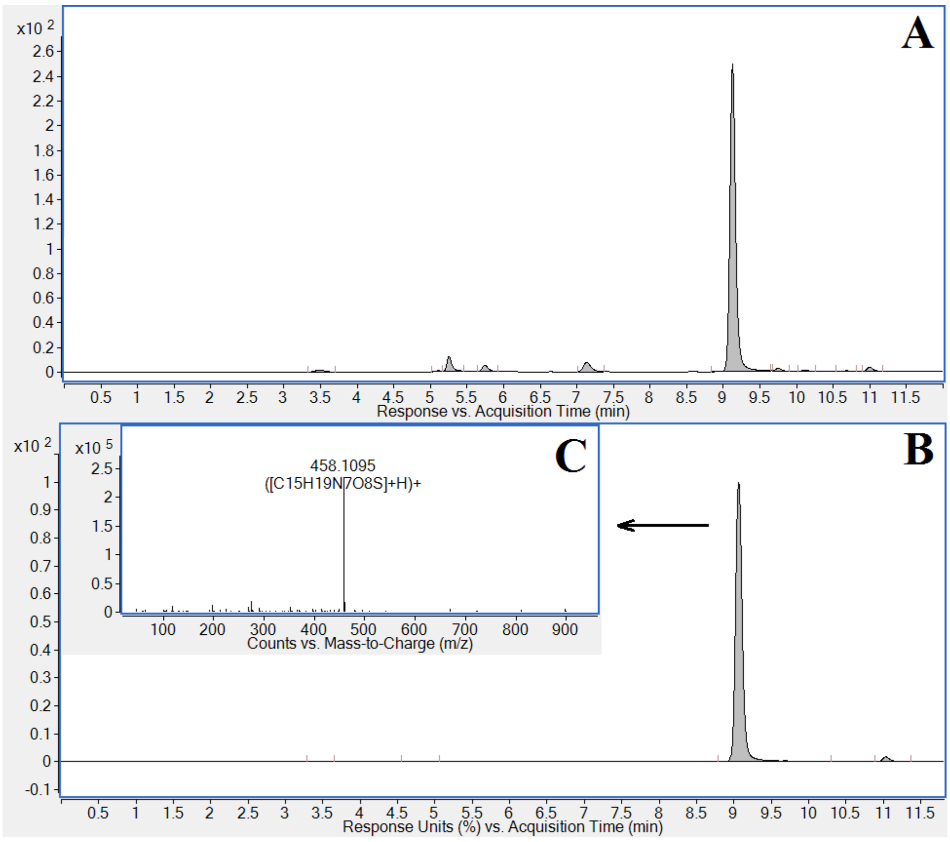

Figure S7d. HILIC-DAD-QTOF analysis of product **8**. Purity profile (HILIC-DAD - 254 nm) before (panel A) and after (panel B) semi-preparative chromatography. Panel C – MS spectrum of the desired compound (the major peak from UV chromatogram).

<u>S8</u>. 2,2′-((6-([4-Sulfamoylphenyl]amino)-1,3,5-triazine-2,4-diyl)bis(amino))di(3-hydroxybutanoic acid) **9** and its IR (a), ^{1}H (b), ^{13}C (c) NMR and DAD/MS (d) spectra

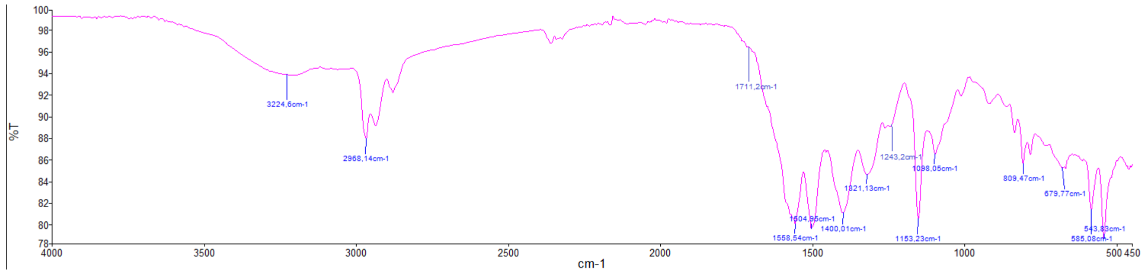

Figure S8a. IR spectra of product **9.**

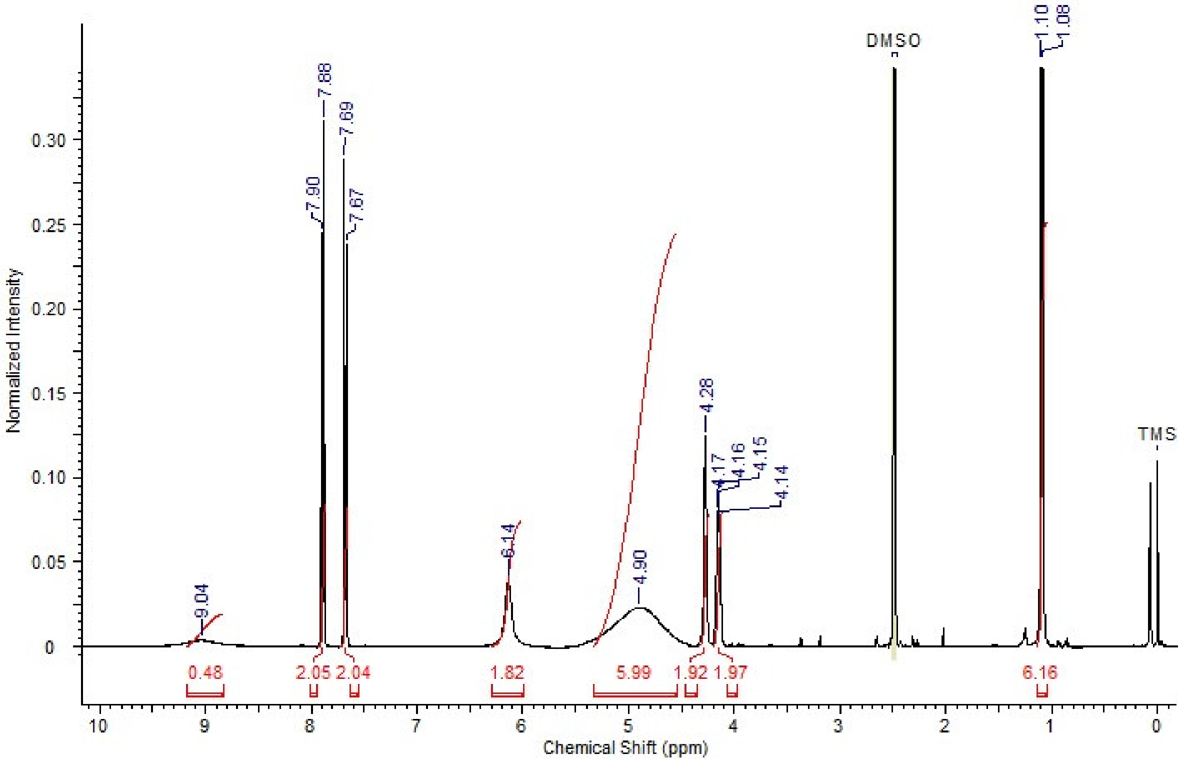

Figure S8b. ^{1}H NMR spectra of product **9.**

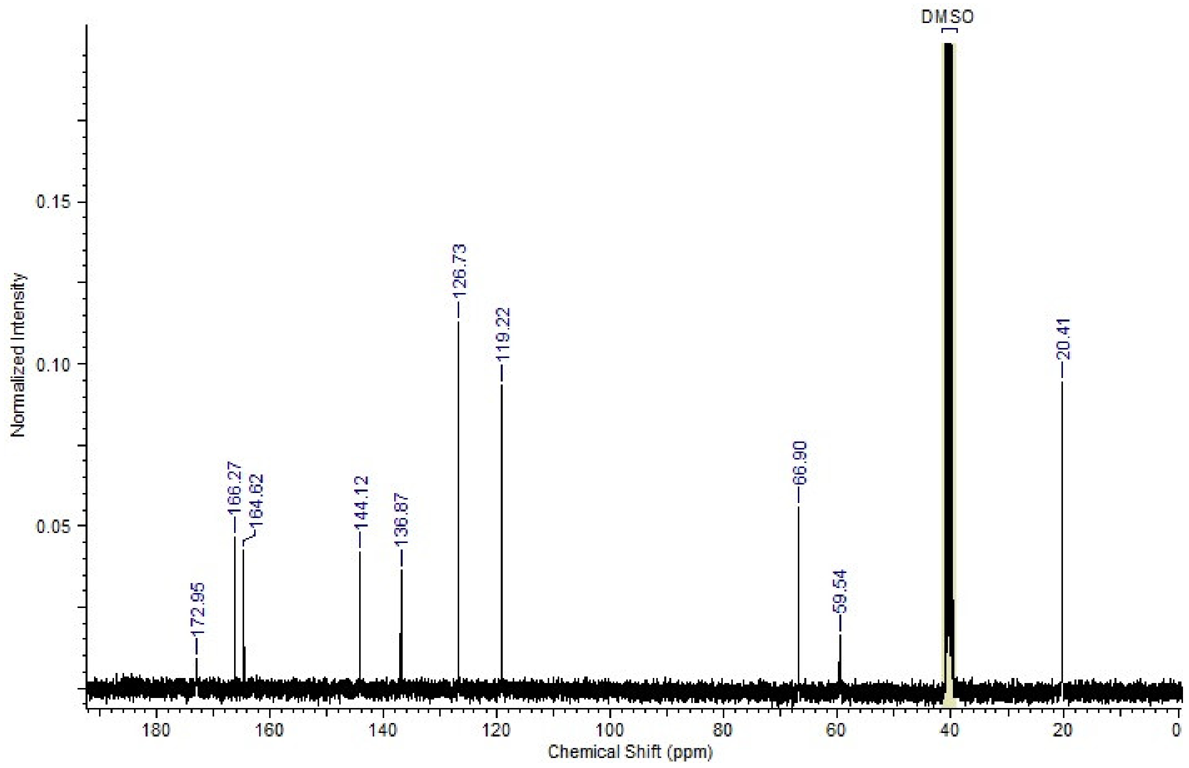

Figure S8c. ^{13}C NMR spectra of product **9.**

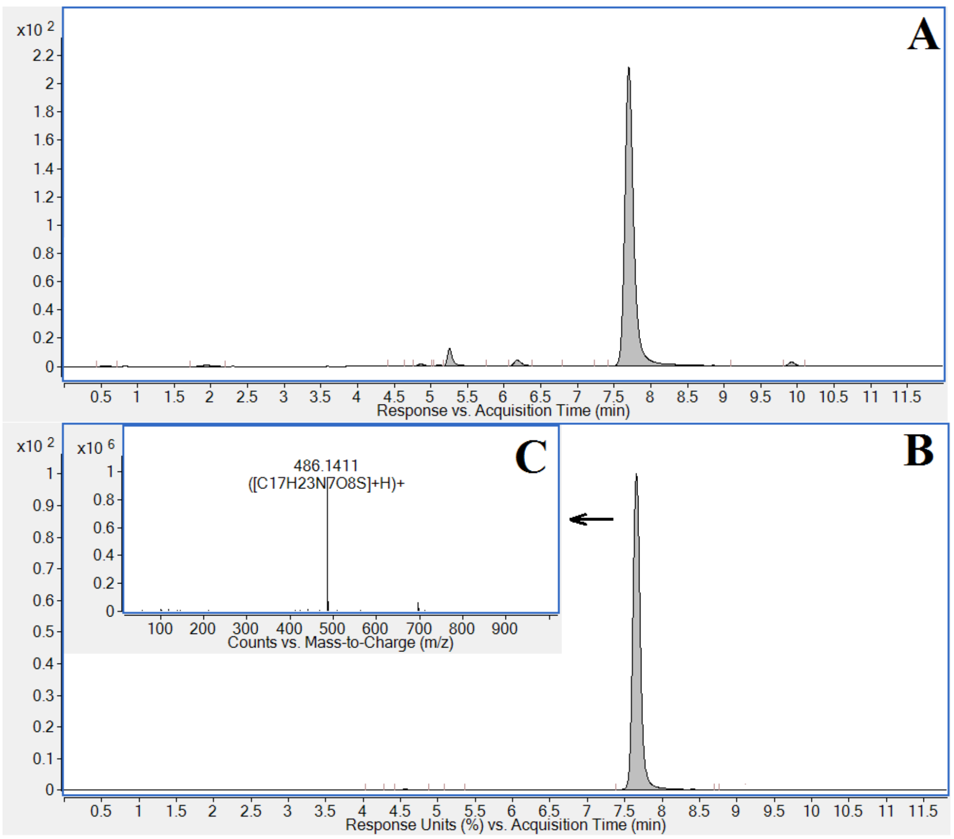

Figure S8d. HILIC-DAD-QTOF analysis of product **9**. Purity profile (HILIC-DAD - 254 nm) before (panel A) and after (panel B) semi-preparative chromatography. Panel C – MS spectrum of the desired compound (the major peak from UV chromatogram).

S9. 2,2′-((6-([4-Sulfamoylphenyl]amino)-1,3,5-triazine-2,4-diyl)bis(amino))disuccinic acid **10** and its IR (a), ^{1}H (b), ^{13}C (c) NMR and DAD/MS (d) spectra.

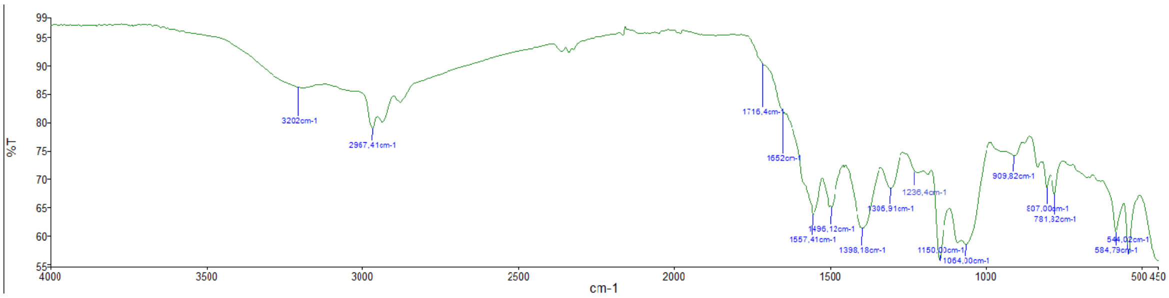

Figure S9a. IR spectra of product **10.**

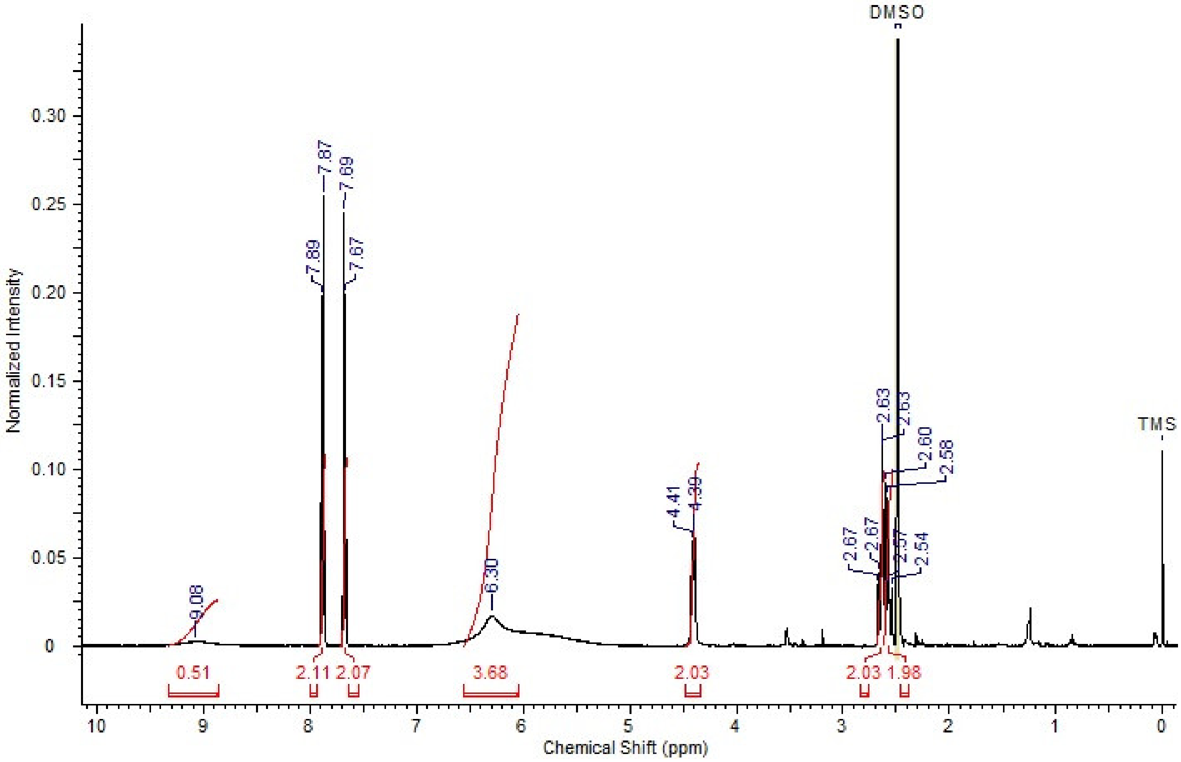

Figure S9b. ^{1}H NMR spectra of product **10.**

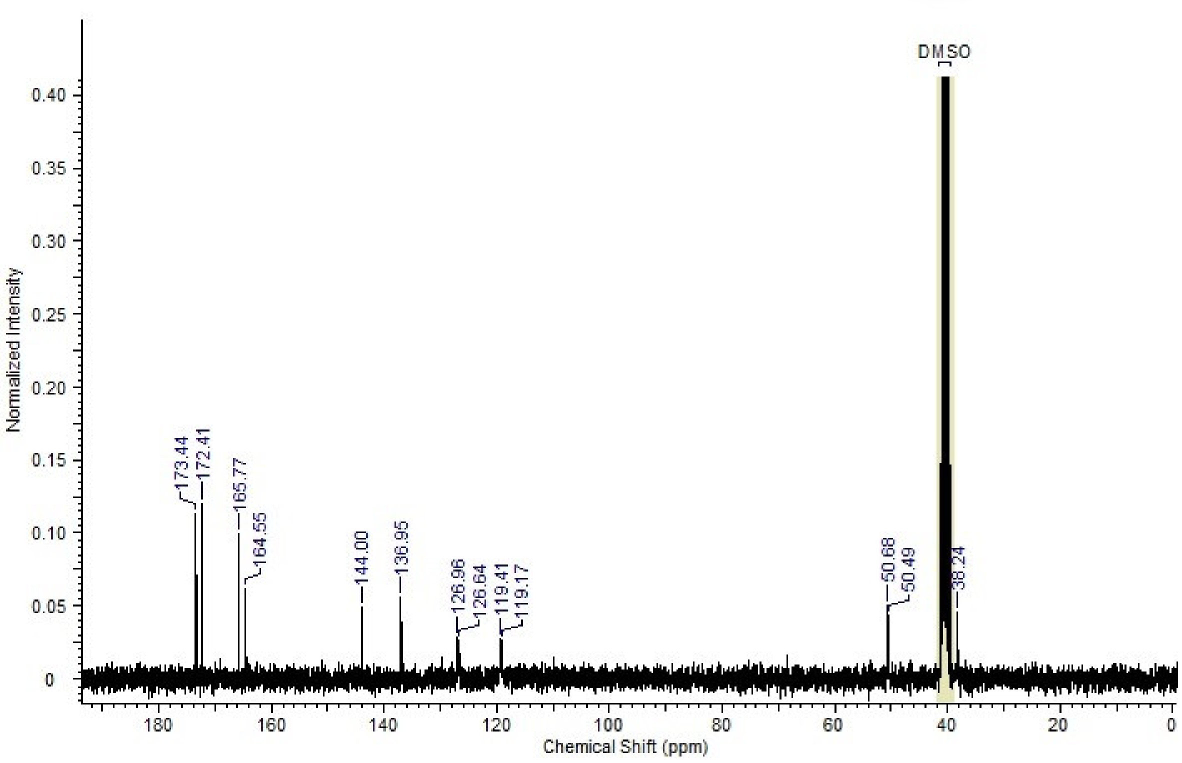

Figure S9c. ^{13}C NMR spectra of product **10.**

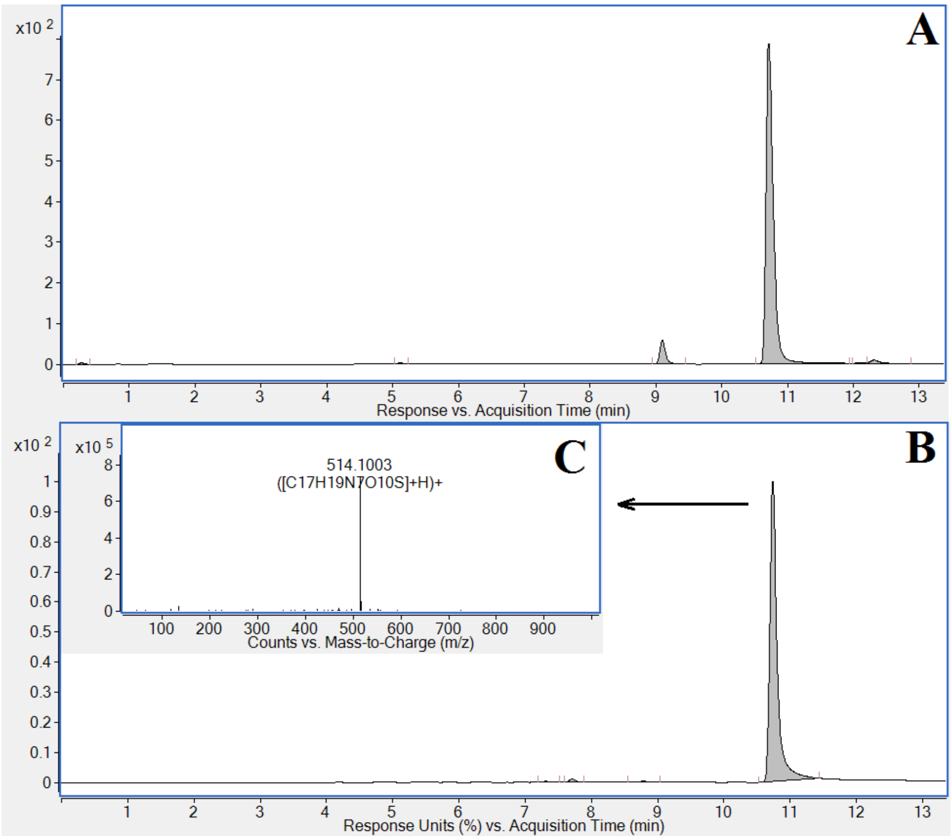

Figure S9d. HILIC-DAD-QTOF analysis of product **10**. Purity profile (HILIC-DAD - 254 nm) before (panel A) and after (panel B) semi-preparative chromatography. Panel C – MS spectrum of the desired compound (the major peak from UV chromatogram).

S10. 2,2′-((6-([4-Sulfamoylphenyl]amino)-1,3,5-triazine-2,4-diyl)bis(amino))diglutaric acid **11** and its IR (a), ^{1}H (b), ^{13}C (c) NMR and DAD/MS (d) spectra

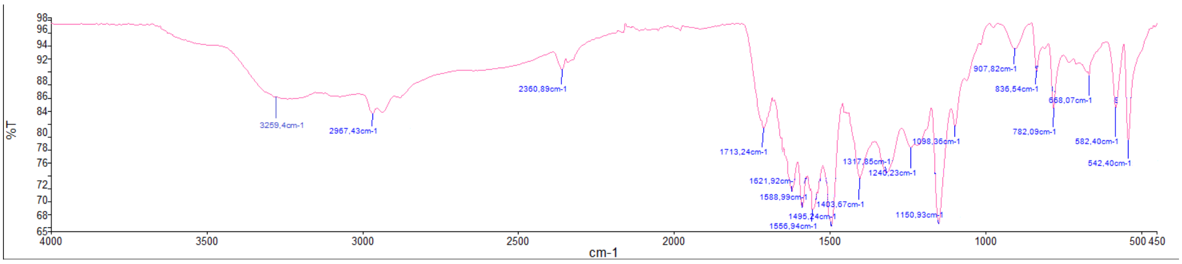

Figure S10a. IR spectra of product **11.**

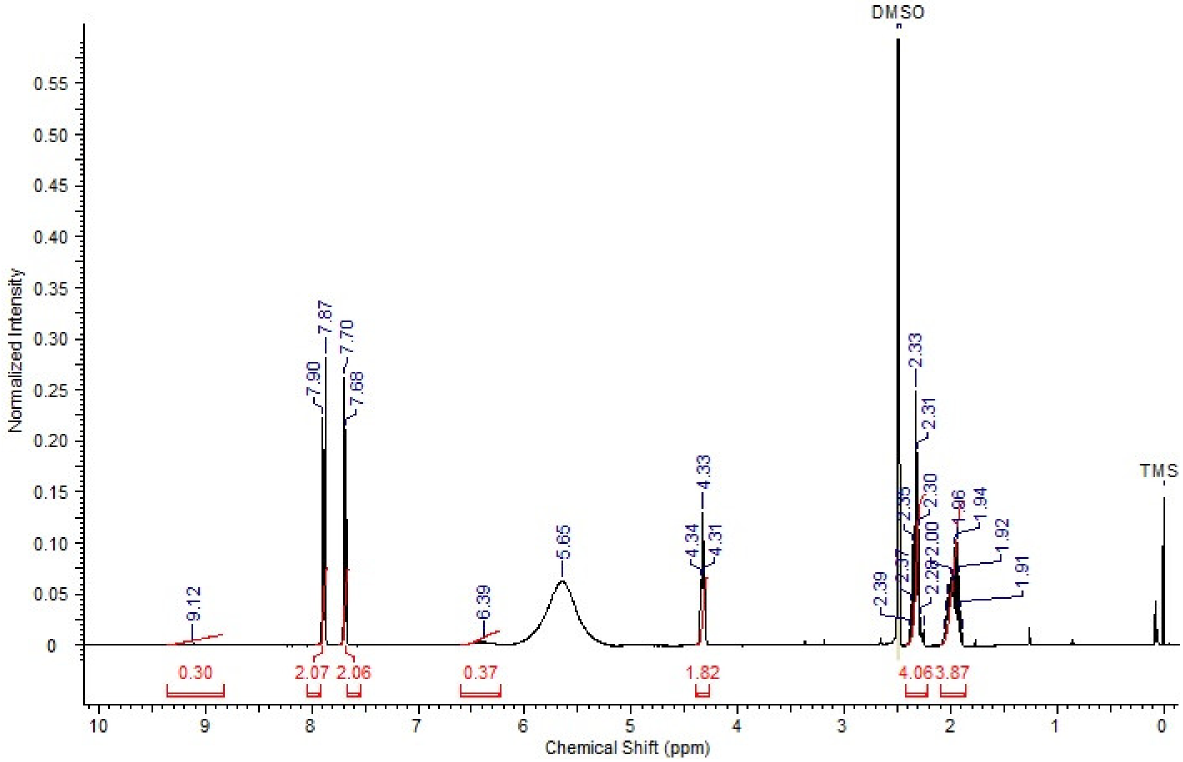

Figure S10b. ^{1}H NMR spectra of product **11.**

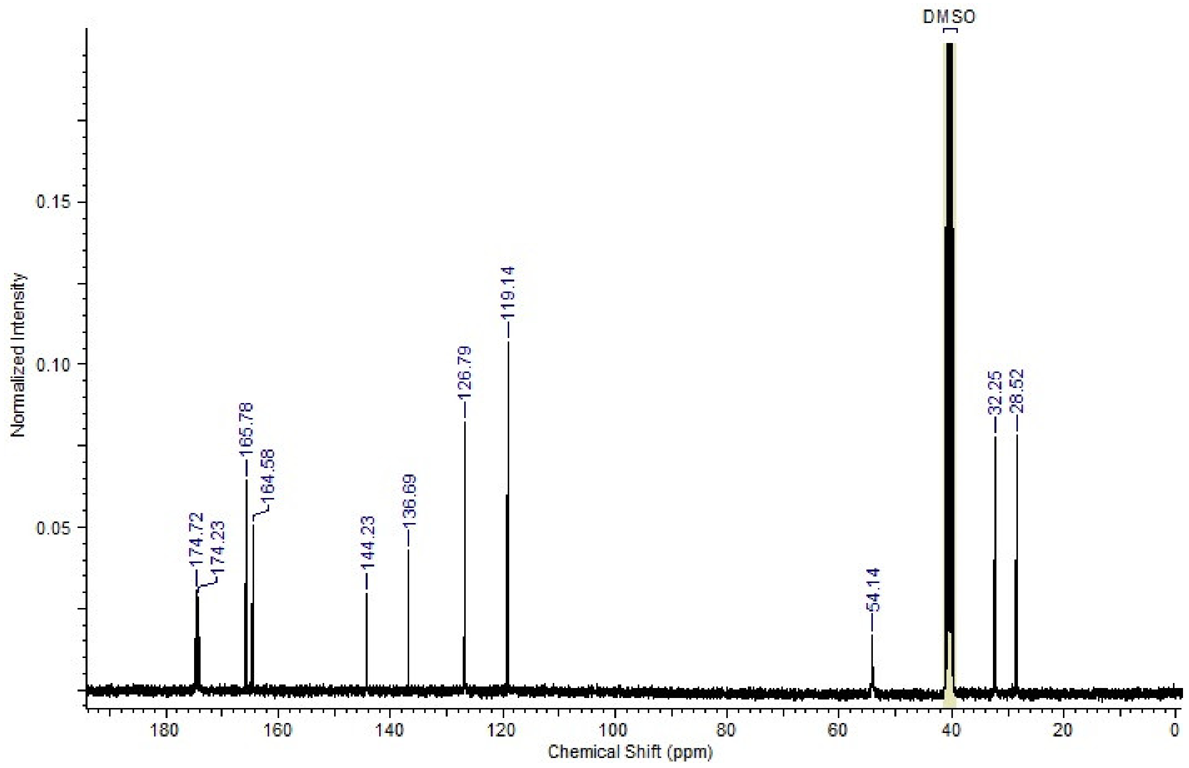

Figure S10c. ^{13}C NMR spectra of product **11.**

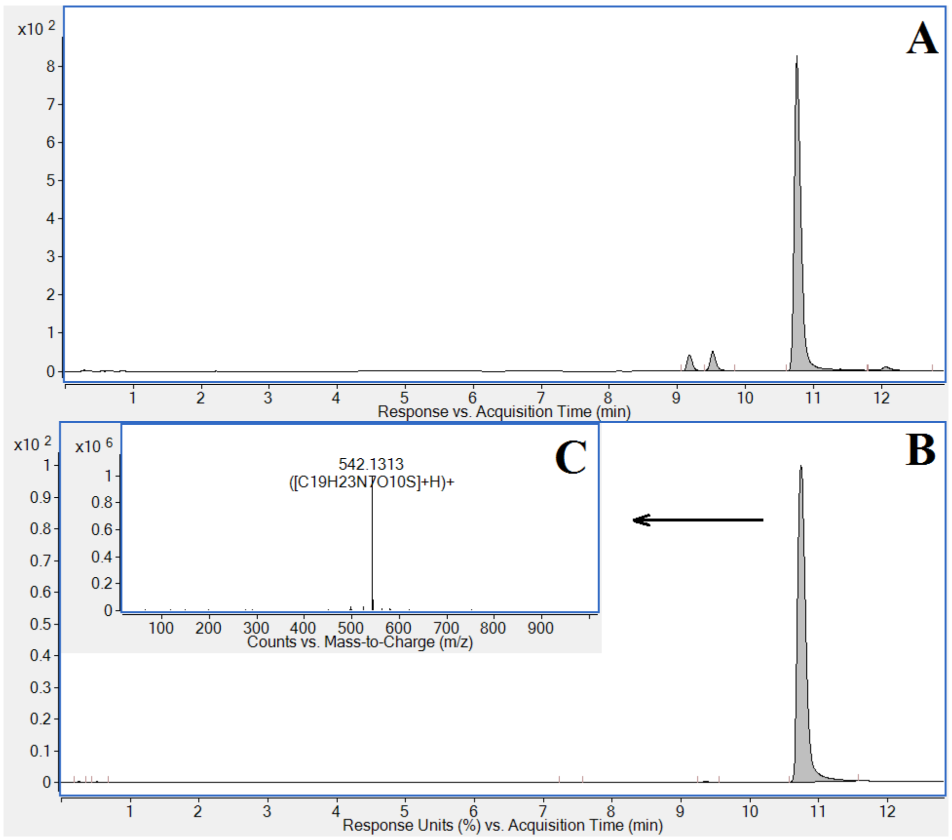

Figure S10d. HILIC-DAD-QTOF analysis of product **11**. Purity profile (HILIC-DAD - 254 nm) before (panel A) and after (panel B) semi-preparative chromatography. Panel C – MS spectrum of the desired compound (the major peak from UV chromatogram).

S11. Selection of molecular descriptors

Examining the molecular descriptors of the model can provide better understanding of the relation between the structure of the derivatives and their inhibitory capability against various hCAs. The values of inhibition against hCA I correlated mostly with *Edge adjacency indices* (7), namely SM05_EA(dm), SM09_EA(dm), SM07_EA(dm), SM10_AEA(ed), SM11_EA(dm), SM08_AEA(ed), and SM09_AEA(ed); *Burden eigenvalues* (5), such as SpMax5_Bh(s), SpMax4_Bh(s), SpMax7_Bh(s), SpMax8_Bh(s), and SpMax6_Bh(s); *2D autocorrelations* descriptor group (4), namely ATSC1s, ATSC6e, ATSC3s, and ATSC4e; *2D matrix-based descriptors* (4), such as SpMAD_H2, EE_B(e), SM3_B(e), and J_D/Dt; *P_VSA-like descriptors* (3), namely P_VSA_s_6, P_VSA_m_3, and P_VSA_e_5; *Constitutional indices* (2 - nO and nDB); GETAWAY descriptors (2 - HATSm and R3s); and 3 descriptors from other groups (*Atom-type E-state indices* - SssCH2, *Geometrical descriptors* - QYYi, and *Information indices* - SIC5). The only selected *Geometrical descriptor*, QYYi, showed highest significance among the selected descriptors according to their *p*-value.

In case of hCA II inhibition, the most influential descriptors belonged to *2D autocorrelations* group (10), namely ATS5p, ATSC7p, ATS7p, ATS8e, ATSC8i, MATS5s, ATS7v, ATS3v, GATS1p, and ATS4p; *2D matrix-based descriptors* (7), specifically SM3_B(m), SM4_B(m), SM5_B(i), SM2_B(s), SM5_B(m), SM4_B(i), and SM5_B(e); *RDF descriptors* (7), namely RDF015u, RDF015m, RDF015v, RDF120i, RDF110v, RDF015p, and RDF120e; *Charge descriptors* (3), such as TE2, PCWTE2 and Qneg; and 3 descriptors from other groups (*Connectivity indices* - X1v, *Topological indices* - ZM1MulPer, and *WHIM descriptors* - G1u).

With respect to the hCA IV, the biggest group of significant variables consisted of *2D autocorrelations* (9), namely ATSC5i, ATSC6i, ATSC7v, ATS1p, ATSC8m, ATS2p, ATSC7i, ATSC1s, and ATSC5p; followed by *Edge adjacency indices* (7), such as Chi0_EA(ed), Chi1_EA(ed), Eig06_EA(dm), SM14_AEA(ed), SM15_AEA(ed), SM02_EA(bo), and Eig02_AEA(ed); *3D-MoRSE descriptors* (4), namely Mor26u, Mor26e, Mor26p, and Mor05u; *2D matrix-based descriptors* (3), such as WiA_B(s), EE_B(s), and SM4_B(e); *3D autocorrelations* (2 - TDB08s and TDB10s); *Charge descriptors* (2 - Qneg and Q2); and 3 descriptors belonging to *Burden eigenvalues* (SpMin3_Bh(s)), *ETA indices* (Eta_beta) or *P_VSA-like descriptors* (P_VSA_LogP_4) group.

Inhibition values against hCA IX correlated mostly with *2D matrix-based descriptors* (7), specifically with SM4_L, SM1_Dz(v), SM5_H2, EE_L, SM5_L, SM1_Dz(i), and SM1_Dz(p); followed by group of *2D matrix-based descriptors* (6), namely P_VSA_s_3, P_VSA_m_4, P_VSA_MR_8, P_VSA_MR_6, P_VSA_LogP_1, and P_VSA_i_1; *2D autocorrelations* (3), such as GATS2m, MATS5m, and GATS4i; *Constitutional indices* (3), such as nHM, H% and nS; *GETAWAY descriptors* (3), namely R1i+, RTi+, and HATS3i; *3D-MoRSE descriptors* (2 - Mor30v and Mor32p); *Edge adjacency indices* (2 - SM02_EA(ri) and SM06_EA(dm)); *Walk and path counts* (2 - MWC06 and SRW06); and 2 descriptors belonging either to *Atom-type E-state indices* (SsCH3) or *Connectivity indices* (X2A) group.

The list of thirty most important molecular descriptors obtained by feature selection in Statistica 12 is in Table ST1. It was found out that a group of 2D autocorrelations as well as 2D matrix-based descriptors appeared to be most significant with respect to inhibitory activity of the sulfonamide derivatives against the all selected human carbonic anhydrases.

Table ST2. The list of thirty most important molecular descriptors obtained by feature selection in Statistica 12 (sorted by p).

	HCA1			HCA2			HCA4			HCA9		
	descriptor	*F-value*	*p-value*	*descriptor*	*F-value*	*p-value*	*descriptor*	*F-value*	*p-value*	*descriptor*	*F-value*	*p-value*
1	QYYi	7,3116	0,0001	ATS5p	11,0889	0,0000	Chi0_EA(ed)	28,8598	0,0000	SM5_H2	11,2717	0,0001
2	ATSC6e	7,3167	0,0002	ATSC7p	9,5183	0,0000	ATSC7v	9,5817	0,0000	Mor30v	7,0422	0,0002
3	SIC5	7,0046	0,0002	SM3_B(m)	9,3779	0,0000	WiA_B(s)	8,5311	0,0000	P_VSA_s_3	8,2497	0,0002
4	HATSm	6,4293	0,0003	SM4_B(m)	9,3765	0,0000	ATS1p	8,0214	0,0001	GATS2m	8,1202	0,0002
5	ATSC3s	8,9179	0,0003	ATS7p	8,5800	0,0000	Chi1_EA(ed)	13,9609	0,0001	MATS5m	6,5890	0,0003
6	SpMax5_Bh(s)	15,7803	0,0005	ATS8e	8,3655	0,0000	Mor26u	8,3370	0,0001	R1i+	6,5433	0,0003
7	SpMax4_Bh(s)	15,7803	0,0005	SM5_B(i)	8,6997	0,0001	Eig06_EA(dm)	8,2965	0,0002	RTi+	6,5433	0,0003
8	P_VSA_s_6	15,7803	0,0005	SM5_B(m)	9,0580	0,0001	SM14_AEA(ed)	9,4207	0,0002	H%	6,2709	0,0003
9	SpMax7_Bh(s)	15,7803	0,0005	TE2	9,7554	0,0001	SM15_AEA(ed)	9,4207	0,0002	GATS4i	6,1352	0,0004
10	nO	15,7803	0,0005	PCWTE2	9,7554	0,0001	ATSC8m	6,5963	0,0002	P_VSA_m_4	16,1143	0,0004
11	SpMax8_Bh(s)	15,7803	0,0005	SM4_B(i)	7,1348	0,0002	TDB08s	6,9606	0,0003	nHM	16,1143	0,0004
12	SM11_EA(dm)	15,7803	0,0005	RDF015u	6,3052	0,0003	P_VSA_LogP_4	8,9909	0,0003	P_VSA_i_1	16,1143	0,0004
13	P_VSA_m_3	15,7803	0,0005	GATS1p	7,6810	0,0004	ATS2p	6,4124	0,0003	nS	16,1143	0,0004
14	SpMax6_Bh(s)	15,7803	0,0005	ATS4p	6,0580	0,0004	ATSC7i	6,4584	0,0003	P_VSA_MR_8	16,1143	0,0004
15	SM05_EA(dm)	15,7803	0,0005	ATSC8i	6,0386	0,0004	Eta_beta	8,7581	0,0003	MWC06	7,1681	0,0005
16	P_VSA_e_5	15,7803	0,0005	G1u	6,1782	0,0004	SM4_B(e)	6,3841	0,0004	SM02_EA(ri)	6,1839	0,0006
17	SM09_EA(dm)	15,7803	0,0005	RDF015m	5,4975	0,0008	Mor26e	6,5431	0,0004	SM4_L	6,5256	0,0006
18	SM07_EA(dm)	15,7803	0,0005	SM2_B(s)	5,9756	0,0010	SM02_EA(bo)	6,9030	0,0004	SRW06	6,1308	0,0006
19	nDB	15,7803	0,0005	RDF015v	5,2214	0,0011	Mor26p	6,4661	0,0004	Mor32p	6,0936	0,0006
20	SpMAD_H2	6,7328	0,0005	RDF120i	5,6287	0,0014	SpMin3_Bh(s)	7,4305	0,0004	EE_L	5,4618	0,0012
21	ATSC1s	8,1282	0,0006	SM5_B(e)	5,5330	0,0016	ATSC1s	8,4014	0,0005	HATS3i	5,0867	0,0013
22	EE_B(e)	5,8219	0,0007	ZM1MulPer	5,1973	0,0017	EE_B(s)	10,2842	0,0005	SM5_L	5,6515	0,0014
23	SM3_B(e)	5,8219	0,0007	MATS5s	4,9765	0,0017	Qneg	7,2720	0,0005	X2A	5,1785	0,0017
24	R3s	5,7348	0,0007	RDF110v	5,4295	0,0018	ATSC5p	6,0413	0,0005	P_VSA_MR_6	8,0756	0,0018
25	SssCH2	5,6636	0,0008	RDF015p	4,7808	0,0019	Eig02_AEA(ed)	7,0593	0,0006	P_VSA_LogP_1	7,9190	0,0020
26	SM10_AEA(ed)	5,8418	0,0008	ATS7v	4,7827	0,0022	TDB10s	6,4828	0,0006	SsCH3	7,9190	0,0020
27	SM08_AEA(ed)	5,8418	0,0008	Qneg	5,6513	0,0022	Mor05u	5,8840	0,0006	SM1_Dz(i)	7,8971	0,0020
28	SM09_AEA(ed)	5,8418	0,0008	RDF120e	4,8217	0,0025	ATSC5i	5,8447	0,0006	SM1_Dz(p)	7,8971	0,0020
29	J_D/Dt	6,1034	0,0009	ATS3v	4,6015	0,0027	ATSC6i	5,8203	0,0007	SM1_Dz(v)	7,8971	0,0020
30	ATSC4e	6,0330	0,0009	X1v	4,5865	0,0027	Q2	6,9602	0,0007	SM06_EA(dm)	7,8971	0,0020
